# Long Short-Term Memory Neural Networks for Modeling Dynamical Processes and Predictive Control: A Hybrid Physics-Informed Approach

**DOI:** 10.3390/s23218898

**Published:** 2023-11-01

**Authors:** Krzysztof Zarzycki, Maciej Ławryńczuk

**Affiliations:** Institute of Control and Computation Engineering, Faculty of Electronics and Information Technology, Warsaw University of Technology, ul. Nowowiejska 15/19, 00-665 Warsaw, Poland; maciej.lawrynczuk@pw.edu.pl

**Keywords:** dynamical systems, LSTM neural networks, physics-informed neural networks, model predictive control

## Abstract

This work has two objectives. Firstly, it describes a novel physics-informed hybrid neural network (PIHNN) model based on the long short-term memory (LSTM) neural network. The presented model structure combines the first-principle process description and data-driven neural sub-models using a specialized data fusion block that relies on fuzzy logic. The second objective of this work is to detail a computationally efficient model predictive control (MPC) algorithm that employs the PIHNN model. The validity of the presented modeling and MPC approaches is demonstrated for a simulated polymerization reactor. It is shown that the PIHNN structure gives very good modeling results, while the MPC controller results in excellent control quality.

## 1. Introduction

Model predictive control (MPC) algorithms, as highlighted in [1,2], find their primary applications in managing processes that classical control methods struggle to handle effectively. These processes often involve multiple-input, multiple-output (MIMO) systems or exhibit strong nonlinearity. MPC, renowned for its flexibility in accommodating various constraints, excels in ensuring high-quality control, even in the face of challenging processes. Real-world instances of successful MPC applications include control of chemical reactors [3,4] and distillation towers [5], as well as the integration of MPC in embedded systems controlling heating ventilation and air conditioning systems (HVAC) [6], quadrotors [7], fuel cells [8], autonomous vehicles [9], and underwater vehicles [10].

As emphasized in [11,12,13], accurate sensor measurements of essential process variables play a critical role in MPC. It is widely acknowledged that the absence of these measurements inevitably leads to a significant loss in control performance. To address this challenge, when the necessary measurements are not readily available, engineers commonly employ online estimation techniques, such as Kalman or extended Kalman filters ([14]). Furthermore, specialized methods and strategies have been developed to tackle this issue in specific applications. In the domain of vehicles, innovative solutions have emerged. The authors of [15] introduce a real-world example where a vehicle employs an external camera to detect obstacles and lane positions on the road. Additionally, it utilizes external rear-corner radars to identify objects approaching from the rear. An intriguing application of sensors is presented in [16], where an anemometer measures external factors such as wind force and direction. Beyond the automotive sector, there are applications like sea ship depth measurement. In [10], a depth sensor is installed for precisely measuring sea ship depth, with heave speed derived from the depth sensor data. Finally, MPC is also used to manage fault-tolerant control. This application addresses issues like stiction in control valves, as discussed in [17].

The cornerstone of any effective MPC algorithm is the precision of its process model. Broadly, two general model classes are usually considered: first-principle (FP) models rooted in the fundamental understanding of the process; black-box approximations. Both model classes have their distinct strengths and limitations:

- FP models demand meticulous process descriptions and accurate parameter values but offer unparalleled modeling precision across a wide operating range, even in abnormal situations. In practice, however, the values of some model parameters may be imprecise or unknown.
- In contrast, data-driven black-box models, including support-vector machines (SVM) [18], multi-layer perceptron (MLP) neural networks [19,20], radial basis function (RBF) networks [21], and recurrent long short-term memory networks (LSTM) [22,23], require no prior domain expertise. Among these, gated recurrent unit (GRU) has gained traction in modeling dynamic systems [24,25] and integrating with MPC algorithms [23,26,27]. Neural networks models have proven to be very useful, especially when dealing with complex dynamical processes, such as predator–prey systems [28,29,30]. However, black-box models may struggle when the available dataset lacks coverage for certain process variables, particularly those operating at infrequent points.

Physics-informed neural network models (PINNs) offer a compelling fusion of both modeling approaches. These models combine the foundational principles governing the process with the data-driven power of machine learning. The result is a versatile model that adheres to fundamental laws while approximating the behavior of real-world processes. The literature showcases PINN applications in scenarios where parameters of ordinary differential equations (ODE) models are either imprecisely known [31] or immeasurable [32]. Furthermore, PINNs can approximate parameters of partial differential equations (PDE) [33]. These PINN models find utility in replacing numerical solvers for ODEs [34] and even serve as models within MPC frameworks [35]. Additionally, one can find several hybrid models aiming to combine a data-driven modeling approach with knowledge of physics. The hybrid physical guided neural network [36] is a feed-forward neural network integrated with a first-principles model. The entire hybrid model is trained jointly. This training process involves incorporating a fusion output layer that utilizes a straightforward interpolation technique. Other examples include using deep neural networks in a physically guided modeling approach [37], in modeling lithium batteries [38], and in modeling a traffic state [39]. One can also find examples of introducing physics directly in the forward pass of the neural network to model the lake temperature [40].

This study addresses a common modeling challenge characterized by two specific limitations. Firstly, process-variable measurements are typically feasible but confined to a limited vicinity of certain operating points. Consequently, the resulting models exhibit localized validity, restricted to the regions where data have been collected for identification purposes. Secondly, although fundamentally sound, the existing first-principle models describing the process often lack precision due to imprecise parameters. In response to these limitations, this work introduces an innovative physics-informed hybrid neural network (PIHNN) model structure, leveraging LSTM neural networks. This approach combines elements from both first-principle and black-box data-driven methodologies, offering robust modeling capabilities in scenarios characterized by the aforementioned issues. Within this research, we delve into two data fusion techniques, drawing from the principles of the first-principle process description and the LSTM network, both employing a fuzzy-logic-based approach. The initial method employs a simplified data fusion block, while the subsequent method harnesses machine learning techniques to minimize overall model errors. To assess the effectiveness of the proposed model structure and data fusion techniques, we apply them to a benchmark polymerization reactor process.

Additionally, we integrate the developed PIHNN model into the MPC framework. Our analysis encompasses a straightforward MPC algorithm with a nonlinear optimization MPC (MPC-NO) and a more intricate linearization-based MPC scheme named the MPC algorithm with nonlinear prediction and linearization around the predicted trajectory (MPC-NPLPT), which relies on computationally uncomplicated quadratic optimization tasks. Our findings demonstrate that the linearization-based MPC approach can yield commendable control performance while significantly reducing computational demands compared to nonlinear counterparts. An initial iteration of the PIHNN model was introduced in conference proceedings [41], where a basic GRU neural network was employed. This current study represents a substantial expansion of previous research efforts. Here, we consider more general LSTM-based PIHNN models, comprehensively examine the model’s structure, explore various potential variants and present details. Furthermore, we introduce an efficient model predictive control (MPC) algorithm for the PIHNN models considered in this study.

This work is organized as follows. Firstly, Section 2 presents the general structure and the details of the hybrid PIHNN model structure utilizing LSTM neural networks. The state–space modeling approach is employed. Secondly, Section 3 briefly describes the general MPC scheme with nonlinear optimization and presents general formulation, necessary implementation details, and the resulting quadratic optimization task of the linearization-based MPC method. Section 4 thoroughly studies the validity of as many as sic PIHNN model variants applied to approximate the behavior of a chemical reactor benchmark. Furthermore, the control efficiency and computational speed of the recommenced linearization-based MPC algorithm is shown. Finally, Section 5 concludes the article.

## 2. Hybrid Physics-Informed Models Using LSTM Neural Networks

We introduce an innovative PIHNN model that blends a data-driven approach with expert knowledge of the underlying physics of the process. To effectively apply the PIHNN model, the following conditions must be satisfied:

- The process input and output variables, i.e., the manipulated and controlled variables, respectively, must be measurable. State variables may be measured or observed using a state estimation, e.g., in the form of an extended Kalman filter (EKF).
- The FP process should exist in the form of a set of differential equations and, when necessary, additional algebraic relations based on the fundamental laws of physics governing the process.

However, we assume that the measurements and the FP model may exhibit imperfections. Specifically, the measurements may originate from a limited range within the entire spectrum of process variable variability. Furthermore, the FP model may also contain inaccuracies and be susceptible to errors arising from factors such as incorrect estimation of specific process parameters or measurement inaccuracies.

### 2.1. Model Structure

This paper primarily focuses on single-input single-output (SISO) process modeling. The process input and output are denoted as *u* and *y*, respectively. Additionally, the process has $n_{x}$ state variables, represented as $x={\left[x_{1}\ldots x_{n_{x}}\right]}^{T}$.

**Figure 1:**
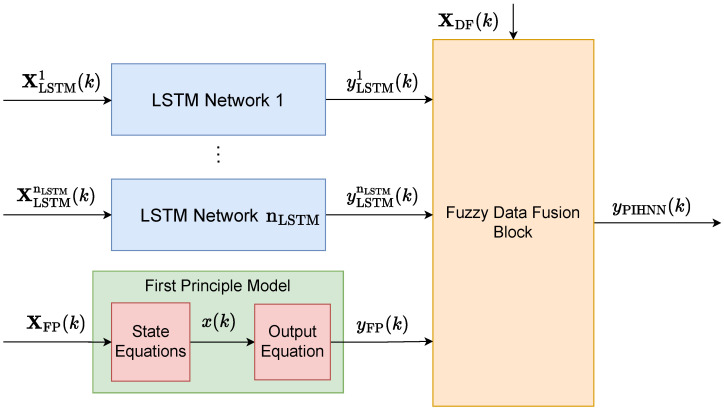
General structure of the PIHNN model.

Figure 1 illustrates the model’s overall structure. The PIHNN model is divided into three distinct components. The first model component, highlighted in blue, is entirely data-driven. It comprises $n_{\mathrm{LSTM}}$ neural sub-models, each trained on available data. The number of data-driven sub-models corresponds to the number of distinct operational areas of the process from which measurement data can be collected. Each sub-model takes the vector $X_{\mathrm{LSTM}}^{i}$ as the input and generates the scalar $y_{\mathrm{LSTM}}^{i}$ as the output. LSTM networks are employed in this study, as earlier research has demonstrated their exceptional ability to model dynamical processes [23,27]. However, it is important to note that alternative data-driven models could also be applied in this context. The second component of the PIHNN structure, highlighted in green, is rooted in expert knowledge about the underlying physics of the process. It consists of an FP sub-model formulated using ordinary differential equations. The input to this sub-model is the vector $X_{\mathrm{FP}}$, while the output is denoted by the scalar $y_{\mathrm{FP}}$. The third component of the PIHNN structure, highlighted in orange, represents the data fusion block (DF). In general, many decision models can be used here, such as neural networks of various architectures. However, we recommend using the fuzzy data fusion block (Fuzzy DF) because it directly incorporates the sub-models. There is no need to train Fuzzy DF on data, which is particularly useful when training data is lacking across specific ranges of process variable variability. By selecting membership function shapes, one can determine which areas and to what extent we should consider the sub-models when calculating the overall PIHNN model output. The DF block takes output calculated by all LSTM sub-models and the FP models as inputs. Based on the current operating state of the process, represented by the vector $X_{\mathrm{DF}}$, it makes decisions regarding the combination of outputs from all sub-models. The primary goal of this fusion process is to minimize the overall error of the entire PIHNN model.

### 2.2. First-Principle Sub-Model

Typically, the FP model utilizes fundamental physical laws formulated in the continuous-time domain, i.e., a set of differential equations must be considered. The state equations have the classical form

(1) $$\begin{array}{cc} {\dot{x}}_{1}\left(t\right) & =f_{1}\left(x_{1}\left(t\right),\ldots ,x_{n_{x}}\left(t\right),u\left(t\right)\right) \end{array}$$

⋮

(2) $$\begin{array}{cc} {\dot{x}}_{n_{x}}\left(t\right) & =f_{n_{x}}\left(x_{1}\left(t\right),\ldots ,x_{n_{x}}\left(t\right),u\left(t\right)\right) \end{array}$$

while the output equation is

(3) $$\begin{array}{cc} y(t) & =g(x_{1}\left(t\right),\ldots ,x_{n_{x}}\left(t\right)) \end{array}$$

where $f_{1},\cdots ,f_{n_{x}}:R^{n_{x}+1}\to R$ and g:R→R are nonlinear functions. Since we will next use the PIHNN model relying on the FP model in the MPC algorithm with online linearization, we require the functions $f_{1},\cdots ,f_{n_{x}},g$ to be differentiable. From Equations (1)–(3), we can find a corresponding discrete-time FP model

(4) $$\begin{array}{cc} x_{1}\left(k\right) & =f_{1}^{d}\left(x_{1}\left(k-1\right),\ldots ,x_{n_{x}}\left(k-1\right),u\left(k-1\right)\right) \end{array}$$

⋮

(5) $$\begin{array}{cc} x_{n_{x}}\left(k\right) & =f_{n_{x}}^{d}\left(x_{1}\left(k-1\right),\ldots ,x_{n_{x}}\left(k-1\right),u\left(k-1\right)\right) \end{array}$$

(6) $$\begin{array}{cc} y(k) & =g^{d}\left(x_{1}\left(k-1\right),\ldots ,x_{n_{x}}\left(k-1\right)\right) \end{array}$$

where $f_{1}^{d},\cdots ,f_{n_{x}}^{d}:R^{n_{x}+1}\to R$ and $g^{d}:R\to R$ are nonlinear mapping functions. The input vector to the FP model can be expressed as

(7) $$X_{\mathrm{FP}}\left(k\right)={\left[x^{T}\left(k-1\right)\;u\left(k-1\right)\right]}^{T}$$

### 2.3. LSTM Sub-Model

**Figure 2:**
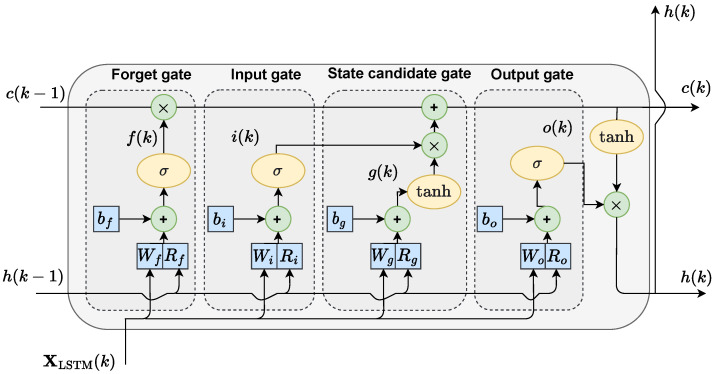
Structure of the LSTM cell.

LSTM networks were developed in response to the vanishing gradient problem that impacts traditional recurrent neural networks [42]. Each LSTM neuron is referred to as a “cell” (Figure 2) and encompasses gates responsible for governing the flow of information within the network. The LSTM cell comprises four distinct gates:

- The forget gate *f* determines which values from the previous cell state should be retained and which should be discarded.
- The input gate *i* selects values from both the previous hidden state and the current input for updating purposes.
- The cell state candidate gate *g* initially regulates the flow of information within the network and subsequently computes the candidate value for the current cell state.
- The output gate *o* is responsible for calculating the new hidden state ‘*h*’.

Each cell in the network has its input vector expressed as

(8) $$X_{\mathrm{LSTM}}\left(k\right)={\left[x_{\mathrm{LSTM}}^{1},\cdots ,x_{\mathrm{LSTM}}^{m}\right]}^{T}={\left[u\left(k-1\right),\cdots ,u\left(k-n_{B}\right),y\left(k-1\right),\cdots ,y\left(k-n_{A}\right)\right]}^{T}$$

where parameters $n_{A}$ and $n_{B}$ define the order of dynamics of the model. The LSTM network has $n_{N}$ cells. The weights in the network can be written in a matrix form

(9) $$W=\left[\begin{array}{c} W_{i}\\ W_{f}\\ W_{g}\\ W_{o} \end{array}\right],\;R=\left[\begin{array}{c} R_{i}\\ R_{f}\\ R_{g}\\ R_{o} \end{array}\right],\;b=\left[\begin{array}{c} b_{i}\\ b_{f}\\ b_{g}\\ b_{o} \end{array}\right]$$

The input weight matrices $W_{i}$, $W_{f}$, $W_{g}$ and $W_{o}$ have dimensionality $n_{N}\times n_{f}$; the recurrent weight matrices $R_{i}$, $R_{f}$, $R_{g}$ and $R_{o}$ have dimensionality $n_{N}\times n_{N}$; and the bias vectors $b_{i}$, $b_{f}$, $b_{g}$ and $b_{o}$ have dimensionality $n_{N}\times 1$, respectively. At time instant *k*, the LSTM model initially calculates the output value of each gate

(10) $$\begin{array}{cc} i(k) & =\sigma \left(W_{i}X_{\mathrm{LSTM}}+R_{i}h\left(k-1\right)+b_{i}\right) \end{array}$$

(11) $$\begin{array}{cc} f(k) & =\sigma \left(W_{f}X_{\mathrm{LSTM}}+R_{f}h\left(k-1\right)+b_{f}\right) \end{array}$$

(12) $$\begin{array}{cc} \;g(k) & =\tanh\left(W_{g}X_{\mathrm{LSTM}}+R_{g}h\left(k-1\right)+b_{g}\right) \end{array}$$

(13) $$\begin{array}{cc} o(k) & =\sigma \left(W_{o}X_{\mathrm{LSTM}}+R_{o}h\left(k-1\right)+b_{o}\right) \end{array}$$

Subsequently, the cell state of the network can be computed

(14) $$\begin{array}{c} c(k)=f(k)\circ c(k-1)+i(k)\circ g(k) \end{array}$$

where the symbol ∘ represents the Hadamard product of vectors. Finally, the hidden state can calculated

(15) $$\begin{array}{c} h(k)=o(k)\circ \tanh(c(k)) \end{array}$$

**Figure 3:**
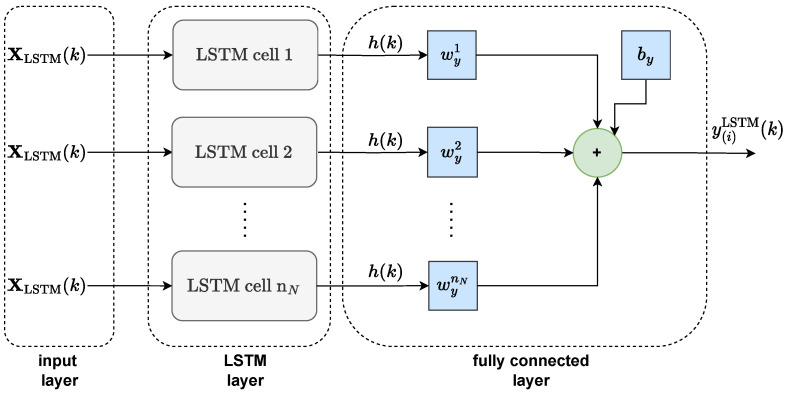
Structure of the whole LSTM network.

The LSTM layer of the network is typically added to a fully connected layer (Figure 3), with weight matrix $W_{y}$ with a dimensionality of $1\times n_{N}$ and bias $b_{y}$. Finally, the computation of the network’s output at time instant *k* can be expressed as

(16) $$\begin{array}{c} y_{\left(i\right)}^{\mathrm{LSTM}}\left(k\right)=W_{y}h\left(k\right)+b_{y} \end{array}$$

One can represent Equations (10)–(15) in scalar form, which will prove useful for the derivation of the MPC algorithm considered in Section 3. The scalar form expressions for the *n*-th elements of the gate and state vectors are

(17) $$\begin{array}{cc} i_{n}\left(k\right) & =\sigma \left(\sum_{m=1}^{n_{A}+n_{B}}\left(w_{n,m}^{i}x_{\mathrm{LSTM}}^{m}\left(k\right)\right)+\sum_{m=1}^{n_{N}}\left(r_{n,m}^{i}h_{m}\left(k-1\right)\right)+b_{n}^{i}\right) \end{array}$$

(18) $$\begin{array}{cc} f_{n}\left(k\right) & =\sigma \left(\sum_{m=1}^{n_{A}+n_{B}}\left(w_{n,m}^{f}x_{\mathrm{LSTM}}^{m}\left(k\right)\right)+\sum_{m=1}^{n_{N}}\left(r_{n,m}^{f}h_{m}\left(k-1\right)\right)+b_{n}^{f}\right) \end{array}$$

(19) $$\begin{array}{cc} g_{n}\left(k\right) & =\tanh\left(\sum_{m=1}^{n_{A}+n_{B}}\left(w_{n,m}^{g}x_{\mathrm{LSTM}}^{m}\left(k\right)\right)+\sum_{m=1}^{n_{N}}\left(r_{n,m}^{g}h_{m}\left(k-1\right)\right)+b_{n}^{g}\right) \end{array}$$

(20) $$\begin{array}{cc} \;o_{n}\left(k\right) & =\sigma \left(\sum_{m=1}^{n_{A}+n_{B}}\left(w_{n,m}^{o}x_{\mathrm{LSTM}}^{m}\left(k\right)\right)+\sum_{m=1}^{n_{N}}\left(r_{n,m}^{o}h_{m}\left(k-1\right)\right)+b_{n}^{o}\right) \end{array}$$

(21) $$\begin{array}{ll} \;c_{n}\left(k\right) & =f_{n}\left(k\right)c_{n}\left(k-1\right)+i_{n}\left(k\right)g_{n}\left(k\right) \end{array}$$

(22) $$\begin{array}{ll} \;h_{n}\left(k\right) & =o_{n}\left(k\right)\tanh\left(c_{n}\left(k\right)\right) \end{array}$$

(23) $$\begin{array}{ll} \;y_{\left(i\right)}^{\mathrm{LSTM}}\left(k\right) & =\sum_{n=1}^{n_{N}}w_{y}^{n}h_{n}\left(k\right)+b_{y} \end{array}$$

Equations (22) and (23) could be used to find the output of the network in the form of one equation

(24) $$\begin{array}{cc} y_{\left(i\right)}^{\mathrm{LSTM}}\left(k\right)= & \sum_{n=1}^{n_{N}}w_{y}^{n}\left(o_{n}\left(k\right)\tanh\left(f_{n}\left(k\right)c_{n}\left(k-1\right)+i_{n}\left(k\right)g_{n}\left(k\right)\right)\right)+b_{y} \end{array}$$

### 2.4. Fuzzy Data Fusion Block

Considering Figure 1, the output of the whole PIHNN model is

(25) $$\begin{array}{c} y_{\mathrm{PIHNN}}\left(k\right)=\frac{\sum_{n=1}^{n_{\mathrm{LSTM}}}y_{n}^{\mathrm{LSTM}}\left(k\right)\mu_{n}^{\mathrm{LSTM}}\left(k\right)+y_{\mathrm{FP}}\left(k\right)\left(\sum_{n=1}^{n_{\mathrm{FP}}}\mu_{n}^{\mathrm{FP}}\left(k\right)\right)}{\sum_{n=1}^{n_{\mathrm{LSTM}}}\mu_{n}^{\mathrm{LSTM}}\left(k\right)+\sum_{n=1}^{n_{\mathrm{FP}}}\mu_{n}^{\mathrm{FP}}\left(k\right)} \end{array}$$

In this study, we use trapezoidal, sigmoidal, and Gaussian membership functions. For trapezoidal functions, we have

(26) $$\mu_{n}^{\mathrm{LSTM}}\left(k\right)=\mu^{\mathrm{LSTM}}\left(X_{\mathrm{DF}}\left(k\right)\right)=\max\left(\min\left(\frac{X_{\mathrm{DF}}\left(k\right)-a_{n}}{b_{n}-a_{n}},1,\frac{d_{n}-X_{\mathrm{DF}}\left(k\right)}{d_{n}-c_{n}}\right),0\right)$$

for sigmoidal ones, we write

(27) $$\mu_{n}^{\mathrm{LSTM}}\left(k\right)=\mu^{\mathrm{LSTM}}\left(X_{\mathrm{DF}}\left(k\right)\right)=\frac{1}{1+e^{-a_{n}\left(X_{\mathrm{DF}}\left(k\right)-b_{n}\right)}}\frac{1}{1+e^{-c_{n}\left(X_{\mathrm{DF}}\left(k\right)-d_{n}\right)}}$$

and for Gaussian ones, we define

(28) $$\mu_{n}^{\mathrm{LSTM}}\left(k\right)=\mu^{\mathrm{LSTM}}\left(X_{\mathrm{DF}}\left(k\right)\right)=\exp\left(\frac{-(X_{\mathrm{DF}}\left(k\right)-a_{n})}{2b_{n}^{2}}\right)$$

The signal $X_{\mathrm{DF}}\left(k\right)=y\left(k\right)$ or $X_{\mathrm{DF}}\left(k\right)=u\left(k-1\right)$ defines the current operating point of the process. Parameters $a_{n}$, $b_{n}$, $c_{n}$, $d_{n}$ define the shape of membership functions used.

### 2.5. Model Development Procedure

The process for establishing the PIHNN model unfolds as follows:

1. We determine the number of distinct training datasets that can be derived from the process measurements.
2. We conduct training of the LSTM network for each training dataset.
3. We implement a discrete FP model of the process.
4. We select the initial shape and range of the membership function within the DF block.
5. We deliver the outputs of the LSTM sub-models and the output of the FP model as inputs of the DF block, where their fusion is carried out based on the current operational state of the process. This fusion process determines the output of the PIHNN model.
6. We assess the quality of PIHNN modeling. If it proves unsatisfactory, then it becomes necessary to modify the shape of the membership function.
7. We adjust the membership function’s shape, which can be executed manually, drawing upon expert knowledge, or using an optimization procedure.

**Figure 4:**
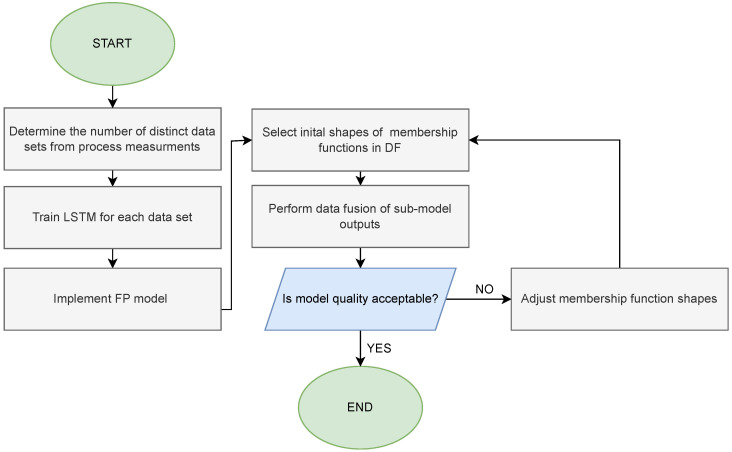
Flow chart for development of PIHNN model.

The flow chart of the model development procedure is also presented in Figure 4.

## 3. LSTM PIHNN Models in Predictive Control

### 3.1. Basic Predictive Control Problem Formulation

This work utilizes the general MPC formulation [1,2]. Namely, at each discrete-time sampling instant *k*, where k=0,1,2,…, the MPC controller performs real-time calculations to determine the vector of decision variables. It is defined as the following current and future increments of the input variable

(29) $$▵u\left(k\right)={\left[▵u\left(k|k\right)\;▵u\left(k+1|k\right)\;\ldots \;▵u\left(k+N_{u}-1|k\right)\right]}^{T}$$

The symbol ▵u(k|k) represents the increment of the manipulated variable at time instant *k*, computed at the same time instant *k*. Similarly, the symbol ▵u(k+1|k) corresponds to the increment of the manipulated variable at the future time instant k+1, computed at the current time instant *k*. This notation extends to subsequent time instants as well. $N_{u}$ represents the control horizon, which determines the length of the MPC decision variable vector. The fundamental MPC optimization problem aims to minimize the predicted control error, minimize excessive increments of the manipulated variable, and satisfy constraints. Let us denote the set-point of the controlled variable for the future sampling instant k+p known at the current instant *k* by $y^{\mathrm{sp}}\left(k+p|k\right)$ and the corresponding prediction determined from the process model by $\hat{y}\left(k+p|k\right)$. We consider the predictions and control errors on the prediction horizon *N*. As far as the magnitude constraints on the manipulated variable and the predicted controlled variable are concerned, they are represented by $u^{\min}$, $u^{\max}$ and $y^{\min}$, $y^{\max}$, respectively. The fundamental MPC optimization task can be formulated as follows:

(30) $$\begin{array}{cc} \; & \min_{▵u(k)}\left\{J\left(k\right)=\sum_{p=1}^{N}\left(y^{\mathrm{sp}}{\left(k+p|k\right)}^{2}-\hat{y}\left(k+p|k\right)\right)+\lambda \sum_{p=0}^{N_{u}-1}{\left(▵u(k+p|k)\right)}^{2}\right\}\\ \; & \mathrm{subject}\;\mathrm{to}\\ \; & u^{\min}\leq u\left(k+p|k\right)\leq u^{\max},\;p=0,\ldots ,N_{u}-1\\ \; & ▵u^{\min}\leq ▵u\left(k+p|k\right)\leq ▵u^{\max},\;p=0,\ldots ,N_{u}-1\\ \; & y^{\min}\leq \hat{y}\left(k+p|k\right)\leq y^{\max},\;p=1,\ldots ,N. \end{array}$$

In general, the predictions over the prediction horizon are obtained as

(31) $$\hat{y}\left(k+p|k\right)=y\left(k+p|k\right)+d\left(k\right)$$

where the model output for the future discrete time k+p, determined at the current time *k*, is denoted as y(k+p|k). The unmeasured disturbance, that covers the model error and real disturbances that act on the controlled process, is computed as the difference between the measured value of the process controlled variable and its estimation obtained from the model. The MPC optimization problem (30) is solved online at each sampling instant, yielding the solution vector (29). According to the principle of repetitive control, the first element of the obtained solution vector is sent to the process and the whole procedure is repeated in the subsequent sampling instants.

### 3.2. Nonlinear MPC Optimization for PIHNN Models

Suppose a nonlinear model, e.g., an LSTM structure or the PIHNN model described in this work, is directly used to determine the predictions $\hat{y}\left(k+p|k\right)$. The general MPC optimization problem (30) becomes nonlinear in that case. We will refer to such a control method as MPC-NO.

### 3.3. Quadratic MPC Optimization for PIHNN Models

In order to derive a computationally attractive alternative to the MPC-NO method, we derive an MPC with successive linearization of the predicted trajectory. Such an approach will make it possible to derive a quadratic optimization MPC task. We use the general approach to predicted trajectory linearization known as the MPC-NPLPT method, introduced in [19,20]. However, the application of an original PIHNN model structure requires careful derivation of the algorithm. Firstly, let us define the predicted trajectory of the controlled variable over the entire prediction horizon, i.e., the following vector:

(32) $$\hat{y}\left(k\right)={\left[\hat{y}\left(k+1|k\right)\;\ldots \;\hat{y}\left(k+N|k\right)\right]}^{T}$$

In the MPC-NPLPT approach, linearization is performed along a trajectory of the manipulated variable defined over the control horizon. It has the following form:

(33) $$u^{\mathrm{traj}}\left(k\right)={\left[u^{\mathrm{traj}}\left(k|k\right)\;\ldots \;u^{\mathrm{traj}}\left(k+N_{u}-1|k\right)\right]}^{T}$$

From the definition of the control horizon, it follows that $u^{\mathrm{traj}}\left(k+p|k\right)=u^{\mathrm{traj}}\left(k+N_{u}-1|k\right)$ for $p=N_{u},\ldots ,N$. The input trajectory (33) is utilized to determine the predicted trajectory of the controlled variable over the prediction horizon

(34) $${\hat{y}}^{\mathrm{traj}}\left(k\right)={\left[{\hat{y}}^{\mathrm{traj}}\left(k+1|k\right)\;\ldots \;{\hat{y}}^{\mathrm{traj}}\left(k+N|k\right)\right]}^{T}$$

For linearization, we use Taylor’s approach. Let us define the vector comprising the current and future values of the manipulated variable that correspond to the MPC decision variable vector (29)

(35) $$u\left(k\right)={\left[u\left(k|k\right)\;\ldots \;u\left(k+N_{u}-1|k\right)\right]}^{T}$$

Taking advantage of the compact vector–matrix notation, the predicted trajectory, $\hat{y}\left(k\right)$, is expressed as the following linear function of the vector (35):

(36) $$\hat{y}\left(k\right)={\hat{y}}^{\mathrm{traj}}\left(k\right)+H\left(k\right)\left(u\left(k\right)-u^{\mathrm{traj}}\left(k\right)\right)$$

The $N\times N_{u}$ matrix

(37) $$H\left(k\right)=\frac{d{\hat{y}}^{\mathrm{traj}}\left(k\right)}{du^{\mathrm{traj}}\left(k\right)}$$

defines partial derivatives of the predicted controlled variable’s trajectory with respect to the future manipulated variable’s trajectory; both trajectories take into account the linearization conditions, so we have to utilize the trajectories ${\hat{y}}^{\mathrm{traj}}\left(k\right)$ and $u^{\mathrm{traj}}\left(k\right)$, respectively. The entries of the matrix H(k) are

(38) $$H_{r+1,p}\left(k\right)=\frac{\partial {\hat{y}}^{\mathrm{traj}}\left(k+p|k\right)}{\partial u^{\mathrm{traj}}\left(k+r|k\right)}$$

for all predictions over the prediction horizon, i.e., p=1,…,N, and all computed values of the manipulated variable over the entire control horizon, i.e., $r=0,\ldots ,N_{u}$. The link between the vectors u(k) and ▵u(k) is

(39) u(k)=J▵u(k)+u(k−1)

when the entries of the $N_{u}\times N_{u}$ auxiliary matrix J are defined as

(40) $$J_{i,j}=\left\{\begin{array}{cc} 0 & \mathrm{if}i<j\\ 1 & \mathrm{if}i\geq j \end{array}\right.$$

and the vector of length $N_{u}$ is

(41) $$u\left(k-1\right)={\left[u\left(k-1\right)\ldots u\left(k-1\right)\right]}^{T}$$

Using the linearized trajectory (36) and the rule (39), the general predictive control optimization task (30) is transformed to the subsequent quadratic optimization problem, as follows:

(42) $$\begin{array}{cc} \; & \min_{▵u(k)}\left\{∥y^{\mathrm{sp}}\left(k\right)-H\left(k\right)J▵u\left(k\right)-\hat{y}\left(k\right)-H\left(k\right)\left(u\left(k-1\right)-u\left(k\right)\right){∥}^{2}+{∥▵u(k)∥}_{\Lambda }^{2}\right\}\\ \; & \mathrm{subject}\;\mathrm{to}\\ \; & u^{\min}\leq J▵u\left(k\right)+u\left(k-1\right)\leq u^{\max}\\ \; & ▵u^{\min}\leq ▵u\left(k\right)\leq ▵u^{\max}\\ \; & y^{\min}\leq H\left(k\right)J▵u\left(k\right)+\hat{y}\left(k\right)+H\left(k\right)\left(u\left(k-1\right)-u\left(k\right)\right)\leq y^{\max} \end{array}$$

The definitions for all necessary symbols used in the above problem are

- Λ: a diagonal $N_{u}\times N_{u}$ matrix with diagonal entries equal to the weighting coefficient λ;
- $u^{\min}$: a vector of length $N_{u}$, where all elements are equal to $u^{\min}$;
- $u^{\max}$: a vector of length $N_{u}$, where all elements are equal to $u^{\max}$;
- $▵u^{\min}$: a vector of length $N_{u}$, where all elements are equal to $▵u^{\min}$;
- $▵u^{\max}$: a vector of length $N_{u}$, where all elements are equal to $▵u^{\max}$;
- $y^{\min}$: a vector of length *N*, where all elements are equal to $y^{\min}$;
- $y^{\max}$: a vector of length *N*, where all elements are equal to $y^{\max}$.

### 3.4. PIHNN Prediction

Let us now discuss how the PIHNN model discussed in this work is utilized for MPC prediction, i.e., to calculate the predicted trajectory of the controlled variable defined by Equation (34). We use Equation (25) for the future time instant k+p which gives

(43) $$\begin{array}{c} y^{\mathrm{PIHNN}}\left(k+p|k\right)=\frac{\sum_{n=1}^{n_{\mathrm{LSTM}}}y_{n}^{\mathrm{LSTM}}\left(k+p|k\right)\mu_{n}^{\mathrm{LSTM}}\left(k\right)+y_{\mathrm{FP}}\left(k+p|k\right)\left(\sum_{n=1}^{n_{\mathrm{FP}}}\mu_{n}^{\mathrm{FP}}\left(k\right)\right)}{\sum_{n=1}^{n_{\mathrm{LSTM}}}\mu_{n}^{\mathrm{LSTM}}\left(k\right)+\sum_{n=1}^{n_{\mathrm{FP}}}\mu_{n}^{\mathrm{FP}}\left(k\right)} \end{array}$$

Taking advantage of Equation (31), the predictions are, therefore, expressed as

(44) $$\begin{array}{c} {\hat{y}}^{\mathrm{PIHNN}}\left(k+p|k\right)=\frac{\sum_{n=1}^{n_{\mathrm{LSTM}}}y_{n}^{\mathrm{LSTM}}\left(k+p|k\right)\mu_{n}^{\mathrm{LSTM}}\left(k\right)+y_{\mathrm{FP}}\left(k+p|k\right)\left(\sum_{n=1}^{n_{\mathrm{FP}}}\mu_{n}^{\mathrm{FP}}\left(k\right)\right)}{\sum_{n=1}^{n_{\mathrm{LSTM}}}\mu_{n}^{\mathrm{LSTM}}\left(k\right)+\sum_{n=1}^{n_{\mathrm{FP}}}\mu_{n}^{\mathrm{FP}}\left(k\right)}+d\left(k\right) \end{array}$$

where the membership functions are defined by Equations (26), (27) or (28). Let us note that the predicted trajectory from the PIHNN model depends on the trajectories generated by both LSTM and FP sub-models. The disturbance (the prediction error) is determined as the difference between the measured process output and its estimation obtained from the model

(45) $$\begin{array}{c} d\left(k\right)=y\left(k\right)-y^{\mathrm{PIHNN}}\left(k\right) \end{array}$$

where the signal $y^{\mathrm{PIHNN}}\left(k\right)$ is found from Equation (31).

### 3.5. LSTM Model Prediction

For each LSTM sub-model, the calculations start with computing the predicted output of the gates. For this purpose, we use Equations (17)–(20) which yield the following

(46) $$\begin{array}{cc} i_{n}\left(k+p|k\right) & =\sigma \left(\sum_{m=1}^{n_{A}+n_{B}}\left(w_{n,m}^{i}x_{\mathrm{LSTM}}^{m}\left(k+p|k\right)\right)+\sum_{m=1}^{n_{N}}\left(r_{n,m}^{i}h_{m}\left(k-1+p|k\right)\right)+b_{n}^{i}\right) \end{array}$$

(47) $$\begin{array}{cc} f_{n}\left(k+p|k\right) & =\sigma \left(\sum_{m=1}^{n_{A}+n_{B}}\left(w_{n,m}^{f}x_{\mathrm{LSTM}}^{m}\left(k+p|k\right)\right)+\sum_{m=1}^{n_{N}}\left(r_{n,m}^{f}h_{m}\left(k-1+p|k\right)\right)+b_{n}^{f}\right) \end{array}$$

(48) $$\begin{array}{cc} \;g_{n}\left(k+p|k\right) & =\tanh\left(\sum_{m=1}^{n_{A}+n_{B}}\left(w_{n,m}^{g}x_{\mathrm{LSTM}}^{m}\left(k+p|k\right)\right)+\sum_{m=1}^{n_{N}}\left(r_{n,m}^{g}h_{m}\left(k-1+p|k\right)\right)+b_{n}^{g}\right) \end{array}$$

(49) $$\begin{array}{cc} o_{n}\left(k+p|k\right) & =\sigma \left(\sum_{m=1}^{n_{A}+n_{B}}\left(w_{n,m}^{o}x_{\mathrm{LSTM}}^{m}\left(k+p|k\right)\right)+\sum_{m=1}^{n_{N}}\left(r_{n,m}^{o}h_{m}\left(k-1+p|k\right)\right)+b_{n}^{o}\right) \end{array}$$

Let us introduce auxiliary integer variables as follows: $I_{\mathrm{uf}}\left(p\right)=\max\left(\min\left(p,n_{B}\right),0\right)$, $I_{\mathrm{yf}}\left(p\right)=\min\left(p-1,n_{A}\right)$. We can represent gate predictions as

(50) $$\begin{array}{cc} i_{n}\left(k+p|k\right)=\sigma ( & \sum_{m=1}^{I_{\mathrm{uf}}\left(p\right)}w_{n,m}^{i}u\left(k-m+p|k\right)+\sum_{m=I_{\mathrm{uf}}\left(p\right)+1}^{n_{B}}w_{n,I_{\mathrm{uf}}\left(p\right)+m}^{i}u\left(k-m+p\right)\\ \; & +\sum_{m=1}^{I_{\mathrm{yf}}\left(p\right)}w_{n,n_{B}+m}^{i}{\hat{y}}^{\mathrm{LSTM}}\left(k-m+p|k\right)+\sum_{m=I_{\mathrm{yf}}\left(p\right)+1}^{n_{A}}w_{n,I_{\mathrm{yf}}\left(p\right)+m}^{i}y\left(k-m+p\right)\\ \; & +\sum_{m=1}^{n_{N}}r_{n,m}^{i}h_{m}\left(k+p-1|k\right)+b_{n}^{i}) \end{array}$$

(51) $$\begin{array}{cc} f_{n}\left(k+p|k\right)=\sigma ( & \sum_{m=1}^{I_{\mathrm{uf}}\left(p\right)}w_{n,m}^{f}u\left(k-m+p|k\right)+\sum_{m=I_{\mathrm{uf}}\left(p\right)+1}^{n_{B}}w_{n,I_{\mathrm{uf}}\left(p\right)+m}^{f}u\left(k-m+p\right)\\ \; & +\sum_{m=1}^{I_{\mathrm{yf}}\left(p\right)}w_{n,n_{B}+m}^{f}{\hat{y}}^{\mathrm{LSTM}}\left(k-m+p|k\right)+\sum_{m=I_{\mathrm{yf}}\left(p\right)+1}^{n_{A}}w_{n,I_{\mathrm{yf}}\left(p\right)+m}^{f}y\left(k-m+p\right)\\ \; & +\sum_{m=1}^{n_{N}}r_{n,m}^{f}h_{m}\left(k+p-1|k\right)+b_{n}^{f}) \end{array}$$

(52) $$\begin{array}{cc} g_{n}\left(k+p|k\right)=\tanh( & \sum_{m=1}^{I_{\mathrm{uf}}\left(p\right)}w_{n,m}^{g}u\left(k-m+p|k\right)+\sum_{m=I_{\mathrm{uf}}\left(p\right)+1}^{n_{B}}w_{n,I_{\mathrm{uf}}\left(p\right)+m}^{g}u\left(k-m+p\right)\\ \; & +\sum_{m=1}^{I_{\mathrm{yf}}\left(p\right)}w_{n,n_{B}+m}^{g}{\hat{y}}^{\mathrm{LSTM}}\left(k-m+p|k\right)+\sum_{m=I_{\mathrm{yf}}\left(p\right)+1}^{n_{A}}w_{n,I_{\mathrm{yf}}\left(p\right)+m}^{g}y\left(k-m+p\right)\\ \; & +\sum_{m=1}^{n_{N}}\left(r_{n,m}^{g}h_{m}\left(k+p-1|k\right)\right)+b_{n}^{g}) \end{array}$$

and

(53) $$\begin{array}{cc} o_{n}\left(k+p|k\right)=\sigma ( & \sum_{m=1}^{I_{\mathrm{uf}}\left(p\right)}w_{n,m}^{o}u\left(k-m+p|k\right)+\sum_{m=I_{\mathrm{uf}}\left(p\right)+1}^{n_{B}}w_{n,I_{\mathrm{uf}}\left(p\right)+m}^{o}u\left(k-m+p\right)\\ \; & +\sum_{m=1}^{I_{\mathrm{yf}}\left(p\right)}w_{n,n_{B}+m}^{o}{\hat{y}}^{\mathrm{LSTM}}\left(k-m+p|k\right)+\sum_{m=I_{\mathrm{yf}}\left(p\right)+1}^{n_{A}}w_{n,I_{\mathrm{yf}}\left(p\right)+m}^{o}y\left(k-m+p\right)\\ \; & +\sum_{m=1}^{n_{N}}r_{n,m}^{o}h_{m}\left(k+p-1|k\right)+b_{n}^{o}) \end{array}$$

Then, the predicted cell and hidden states can be determined from Equations (21) and (22)

(54) $$\begin{array}{ll} c_{n}\left(k+p|k\right) & =f_{n}\left(k+p|k\right)c_{n}\left(k-1+p|k\right)+i_{n}\left(k+p|k\right)g_{n}\left(k+p|k\right) \end{array}$$

(55) $$\begin{array}{ll} \;h_{n}\left(k+p|k\right) & =o_{n}\left(k+p|k\right)\tanh\left(c_{n}\left(k+p|k\right)\right) \end{array}$$

Let us stress that the above equations have to be used recurrently for p=1,…,N. Finally, the predicted output of the i-th LSTM sub-model can be computed from Equation (23)

(56) $$\begin{array}{cc} {\hat{y}}_{\left(i\right)}^{\mathrm{LSTM}}\left(k+p|k\right)= & \sum_{n=1}^{n_{N}}w_{y}^{n}\left(h_{n}\left(k+p|k\right)\right)+b_{y} \end{array}$$

which can be also expressed as

(57) $$\begin{array}{cc} {\hat{y}}_{\left(i\right)}^{\mathrm{LSTM}}\left(k+p|k\right) & =\sum_{n=1}^{n_{N}}w_{y}^{n}(o_{n}\left(k+p|k\right)\tanh(f_{n}\left(k+p|k\right)c_{n}\left(k+p-1|k\right)\\ \; & \;\;\;+i_{n}\left(k+p|k\right)g_{n}\left(k+p|k\right)))+b_{y}+d\left(k\right) \end{array}$$

### 3.6. FP Model Prediction

Using Equations (4) and (5), we find model states and the output for the future time instant k+1

(58) $$\begin{array}{cc} x_{1}\left(k+p|k\right) & =f_{1}\left(x_{1}\left(k+p-1|k\right),\ldots ,x_{n_{x}}\left(k+p-1|k\right),u\left(k+p-1|k\right)\right) \end{array}$$

⋮

(59) $$\begin{array}{cc} \;x_{n_{x}}\left(k+p|k\right) & =f_{n_{x}}\left(x_{1}\left(k+p-1|k\right),\ldots ,x_{n_{x}}\left(k+p|k\right),u\left(k+p|k\right)\right) \end{array}$$

(60) $$\begin{array}{cc} \;y^{\mathrm{FP}}\left(k+p|k\right) & =g(x_{1}\left(k+p-1|k\right),\ldots ,x_{n_{x}}\left(k+p-1|k\right)) \end{array}$$

To simplify the following calculations, let us start with computing the prediction of the states for the time instant k+p

(61) $$\begin{array}{cc} {\hat{x}}_{1}\left(k+1|k\right) & =f_{1}\left(k+1|k\right)=f_{1}\left(x_{1}\left(k\right),\ldots ,x_{n_{x}}\left(k\right),u\left(k|k\right)\right)+\nu_{1}\left(k\right) \end{array}$$

⋮

(62) $$\begin{array}{cc} {\hat{x}}_{n_{x}}\left(k+1|k\right) & =f_{n_{x}}\left(k+1|k\right)=f_{n_{x}}\left(x_{1}\left(k\right),\ldots ,x_{n_{x}}\left(k\right),u\left(k|k\right)\right)+\nu_{n}\left(k\right) \end{array}$$

From Equation (6), we find the corresponding predicted controlled variable:

(63) $$\begin{array}{cc} {\hat{y}}^{\mathrm{FP}}\left(k+1|k\right) & =g\left(k+1|k\right)+d\left(k\right)=g({\hat{x}}_{1}\left(k+1|k\right),\ldots ,{\hat{x}}_{n_{x}}\left(k+1|k\right))+d\left(k\right) \end{array}$$

Next, we can determine the predictions for the subsequent sampling instants:

(64) $$\begin{array}{cc} {\hat{x}}_{1}\left(k+p|k\right) & =f_{1}\left(k+p|k\right)=f_{1}\left({\hat{x}}_{1}\left(k+p-1|k\right),\ldots ,{\hat{x}}_{n_{x}}\left(k+p-1|k\right),u\left(k+p-1|k\right)\right)+\nu_{1}\left(k\right) \end{array}$$

⋮

(65) $$\begin{array}{cc} {\hat{x}}_{n_{x}}\left(k+p|k\right) & =f_{n_{x}}\left(k+p|k\right)=f_{n_{x}}\left({\hat{x}}_{1}\left(k+p-1|k\right),\ldots ,{\hat{x}}_{n_{x}}\left(k+p-1|k\right),u\left(k+p-1|k\right)\right)+\nu_{n}\left(k\right) \end{array}$$

(66) $$\begin{array}{cc} \;{\hat{y}}^{\mathrm{FP}}\left(k+p|k\right) & =g\left(k+p|k\right)+d\left(k\right)=g({\hat{x}}_{1}\left(k+p|k\right),\ldots ,{\hat{x}}_{n_{x}}\left(k+p|k\right))+d\left(k\right) \end{array}$$

where p=2,⋯,N. The state and output disturbances (prediction errors), respectively, are computed as the measurements compared with the outputs of the corresponding model equations

(67) $$\begin{array}{cc} \nu_{1}\left(k\right) & =x_{1}\left(k\right)-f_{1}\left(x_{1}\left(k-1\right),\ldots ,x_{n_{x}}\left(k-1\right),u\left(k-1\right)\right) \end{array}$$

⋮

(68) $$\begin{array}{cc} \nu_{n}\left(k\right) & =x_{n}\left(k\right)-f_{n}\left(x_{1}\left(k-1\right),\ldots ,x_{n_{x}}\left(k-1\right),u\left(k-1\right)\right) \end{array}$$

(69) $$\begin{array}{cc} \;d(k) & =y\left(k\right)-g(x_{1}\left(k-1\right),\cdots ,x_{n}\left(k-1\right)) \end{array}$$

### 3.7. PIHNN Model Derivatives

The entries of the matrix H(k) (Equation (37)) are computed from Equation (38). Differentiation of Equation (44) yields

(70) $$\begin{array}{c} \frac{\partial {\hat{y}}^{\mathrm{PIHNN}}\left(k+p|k\right)}{\partial u(k+r|k)}=\frac{\sum_{n=1}^{n_{\mathrm{LSTM}}}\frac{\partial {\hat{y}}_{n}^{\mathrm{LSTM}}\left(k+p|k\right)}{\partial u(k+r|k)}\mu_{n}^{\mathrm{LSTM}}\left(k\right)+\frac{\partial {\hat{y}}^{\mathrm{FP}}\left(k+p|k\right)}{\partial u(k+r|k)}\left(\sum_{n=1}^{n_{\mathrm{FP}}}\mu_{n}^{\mathrm{FP}}\left(k\right)\right)}{\sum_{n=1}^{n_{\mathrm{LSTM}}}\mu_{n}^{\mathrm{LSTM}}\left(k\right)+\sum_{n=1}^{n_{\mathrm{FP}}}\mu_{n}^{\mathrm{FP}}\left(k\right)} \end{array}$$

Let us note that the derivatives of the whole PIHNN model depend on the LSTM and FP sub-model derivatives.

### 3.8. LSTM Model Derivatives

Derivatives for LSTM sub-models are calculated by differentiating Equation (57)

(71) $$\begin{array}{c} \frac{\partial {\hat{y}}_{\left(i\right)}^{\mathrm{LSTM}}\left(k+p|k\right)}{\partial u(k+r|k)}=\sum_{n=1}^{n_{N}}w_{n}^{y}\frac{\partial h_{n}\left(k+p|k\right)}{\partial u(k+r|k)} \end{array}$$

For all p=1,…,N and $r=0,\ldots ,N_{u}-1$, the subsequent step involves the application of the chain rule of differentiation. Initially, it is imperative to determine the derivatives of gates *i*, *f*, *g*, and *o*. We proceed to differentiate Equation (50):

(72) $$\begin{array}{cc} \frac{\partial i_{n}\left(k+p|k\right)}{\partial u(k+r|k)} & =i_{n}\left(k+p|k\right)\left(1-i_{n}\left(k+p|k\right)\right)(\sum_{m=1}^{I_{\mathrm{uf}}\left(p\right)}w_{n,m}^{i}\frac{\partial u(k-m+p|k)}{\partial u(k+r|k)}\\ \; & \;+\sum_{m=1}^{I_{\mathrm{yf}}\left(p\right)}w_{n,n_{B}+m}^{i}\frac{\partial {\hat{y}}^{\mathrm{LSTM}}\left(k-m+p|k\right)}{\partial u(k+r|k)}+\sum_{m=1}^{n_{N}}r_{n,m}^{i}\frac{\partial h_{m}\left(k+p-1|k\right)}{\partial u(k+r|k)}) \end{array}$$

Equation (51) gives

(73) $$\begin{array}{cc} \frac{\partial f_{n}\left(k+p|k\right)}{\partial u(k+r|k)} & =f_{n}\left(k+p|k\right)\left(1-f_{n}\left(k+p|k\right)\right)(\sum_{m=1}^{I_{\mathrm{uf}}\left(p\right)}w_{n,m}^{f}\frac{\partial u(k-m+p|k)}{\partial u(k+r|k)}\\ \; & \;+\sum_{m=1}^{I_{\mathrm{yf}}\left(p\right)}w_{n,n_{B}+m}^{f}\frac{\partial {\hat{y}}^{\mathrm{LSTM}}\left(k-m+p|k\right)}{\partial u(k+r|k)}+\sum_{m=1}^{n_{N}}r_{n,m}^{f}\frac{\partial h_{m}\left(k+p-1|k\right)}{\partial u(k+r|k)}) \end{array}$$

from Equation (52), we obtain

(74) $$\begin{array}{cc} \frac{\partial g_{n}\left(k+p|k\right)}{\partial u(k+r|k)} & =\left(1-g_{n}^{2}\left(k+1|k\right)\right)(\sum_{m=1}^{I_{\mathrm{uf}}\left(p\right)}w_{n,m}^{g}\frac{\partial u(k-m+p|k)}{\partial u(k+r|k)}\\ \; & \;+\sum_{m=1}^{I_{\mathrm{yf}}\left(p\right)}w_{n,n_{B}+m}^{g}\frac{\partial {\hat{y}}^{\mathrm{LSTM}}\left(k-m+p|k\right)}{\partial u(k+r|k)}+\sum_{m=1}^{n_{N}}r_{n,m}^{g}\frac{\partial h_{m}\left(k+p-1|k\right)}{\partial u(k+r|k)}) \end{array}$$

Finally, using Equation (53), we derive

(75) $$\begin{array}{cc} \frac{\partial o_{n}\left(k+p|k\right)}{\partial u(k+r|k)} & =o_{n}\left(k+p|k\right)\left(1-o_{n}\left(k+p|k\right)\right)(\sum_{m=1}^{I_{\mathrm{uf}}\left(p\right)}w_{n,m}^{o}\frac{\partial u(k-m+p|k)}{\partial u(k+r|k)}\\ \; & \;+\sum_{m=1}^{I_{\mathrm{yf}}\left(p\right)}w_{n,n_{B}+m}^{o}\frac{\partial {\hat{y}}^{\mathrm{LSTM}}\left(k-m+p|k\right)}{\partial u(k+r|k)}+\sum_{m=1}^{n_{N}}r_{n,m}^{o}\frac{\partial h_{m}\left(k+p-1|k\right)}{\partial u(k+r|k)}) \end{array}$$

The following step involves computing the derivative of the cell state *c* using Equation (54)

(76) $$\begin{array}{cc} \frac{\partial c_{n}\left(k+p|k\right)}{\partial u(k+r|k)} & =\frac{\partial f_{n}\left(k+p|k\right)}{\partial u(k+r|k)}c_{n}\left(k+p-1|k\right)+f_{n}\left(k+p|k\right)\frac{\partial c_{n}\left(k+p-1|k\right)}{\partial u(k+r|k)}\\ \; & \;+\frac{\partial i_{n}\left(k+p|k\right)}{\partial u(k+r|k)}g_{n}\left(k+p|k\right)+i_{n}\left(k+p|k\right)\frac{\partial g_{n}\left(k+p|k\right)}{\partial u(k+r|k)} \end{array}$$

and from Equation (54), we can derive the derivatives of the hidden state *h*

(77) $$\begin{array}{cc} \frac{\partial h_{n}\left(k+p|k\right)}{\partial u(k+r|k)} & =\frac{\partial o_{n}\left(k+p|k\right)}{\partial u(k+r|k)}\tanh(c_{n}\left(k+p|k\right))\\ \; & \;+o_{n}\left(k+p|k\right)\left(1-\tanh^{2}\left(c_{n}\left(k+p|k\right)\right)\right)\frac{\partial c_{n}\left(k+p|k\right)}{\partial u(k+r|k)} \end{array}$$

### 3.9. FP Model Derivatives

We start by finding derivatives of the predicted state variables for the sampling instant k+1. Differentiating Equations (61) and (62), we obtain

(78) $$\begin{array}{c} \begin{array}{cc} \frac{\partial {\hat{x}}_{1}\left(k+1|k\right)}{\partial u(k+r|k)} & =\sum_{i=1}^{n_{x}}\frac{\partial f_{1}\left(x_{1}\left(k\right),\ldots ,x_{n_{x}}\left(k\right),u\left(k|k\right)\right)}{\partial x_{i}\left(k\right)}\frac{\partial x_{i}\left(k\right)}{\partial u(k+r|k)}\\ \; & \;+\frac{\partial f_{1}\left(x_{1}\left(k\right),\ldots ,x_{n_{x}}\left(k\right),u\left(k|k\right)\right)}{\partial u(k|k)}\frac{\partial u(k|k)}{\partial u(k+r|k)} \end{array} \end{array}$$

⋮

(79) $$\begin{array}{c} \begin{array}{cc} \frac{\partial {\hat{x}}_{n_{x}}\left(k+1|k\right)}{\partial u(k+r|k)} & =\sum_{i=1}^{n_{x}}\frac{\partial f_{n_{x}}\left(x_{1}\left(k\right),\ldots ,x_{n_{x}}\left(k\right),u\left(k|k\right)\right)}{\partial x_{i}\left(k\right)}\frac{\partial x_{i}\left(k\right)}{\partial u(k+r|k)}\\ \; & \;+\frac{\partial f_{n_{x}}\left(x_{1}\left(k\right),\ldots ,x_{n_{x}}\left(k\right),u\left(k|k\right)\right)}{\partial u(k|k)}\frac{\partial u(k|k)}{\partial u(k+r|k)} \end{array} \end{array}$$

Knowing that

(80) $$\frac{\partial u^{\mathrm{traj}}\left(k+p|k\right)}{\partial u^{\mathrm{traj}}\left(k+r|k\right)}=\left\{\begin{array}{cc} 1 & \mathrm{if}\;p=r\;\mathrm{or}\;(p>r\;\mathrm{and}\;r=N_{u}-1)\\ 0 & \mathrm{otherwise} \end{array}\right.$$

we can simplify Equations (78) and (79) to

(81) $$\begin{array}{cc} \frac{\partial {\hat{x}}_{1}\left(k+1|k\right)}{\partial u(k+r|k)} & =\frac{\partial f_{1}\left(x_{1}\left(k\right),\ldots ,x_{n_{x}}\left(k\right),u\left(k|k\right)\right)}{\partial u(k|k)}\frac{\partial u(k|k)}{\partial u(k+r|k)} \end{array}$$

⋮

(82) $$\begin{array}{cc} \frac{\partial {\hat{x}}_{n_{x}}\left(k+1|k\right)}{\partial u(k+r|k)} & =\frac{\partial f_{n_{x}}\left(x_{1}\left(k\right),\ldots ,x_{n_{x}}\left(k\right),u\left(k|k\right)\right)}{\partial u(k|k)}\frac{\partial u(k|k)}{\partial u(k+r|k)} \end{array}$$

The next step is to find the derivative for the FP model states and the controlled variable for prediction at the sampling instant k+p, where p=2,…,N. From Equation (63), we have

(83) $$\begin{array}{cc} \frac{\partial {\hat{y}}^{\mathrm{FP}}\left(k+1|k\right)}{\partial u(k+r|k)} & =\sum_{i=1}^{n_{x}}\frac{\partial g({\hat{x}}_{1}\left(k+1|k\right),\ldots ,{\hat{x}}_{n_{x}}\left(k+1|k\right))}{\partial {\hat{x}}_{i}\left(k+1|k\right)}\frac{\partial {\hat{x}}_{i}\left(k+1|k\right)}{\partial u(k+r|k)} \end{array}$$

Next, we can determine the derivatives when p=2,⋯,N. We start with the state variables. From Equations (64) and (65), we obtain

(84) $$\begin{array}{c} \begin{array}{cc} \frac{\partial {\hat{x}}_{1}\left(k+p|k\right)}{\partial u(k+r|k)} & =\sum_{i=1}^{n_{x}}\frac{\partial f_{1}\left({\hat{x}}_{1}\left(k+p-1|k\right),\ldots ,{\hat{x}}_{n_{x}}\left(k+p-1|k\right),u\left(k+p-1|k\right)\right)}{\partial {\hat{x}}_{i}\left(k+p-1|k\right)}\\ \; & \;\;\times \frac{\partial {\hat{x}}_{i}\left(k+p-1|k\right)}{\partial u(k+r|k)}\\ \; & \;+\frac{\partial f_{1}\left({\hat{x}}_{1}\left(k+p-1|k\right),\ldots ,{\hat{x}}_{n_{x}}\left(k+p-1|k\right),u\left(k+p-1|k\right)\right)}{\partial u(k+p-1|k)}\\ \; & \;\times \frac{\partial u(k+p-1|k)}{\partial u(k+r|k)} \end{array} \end{array}$$

⋮

(85) $$\begin{array}{c} \begin{array}{cc} \frac{\partial {\hat{x}}_{n_{x}}\left(k+p|k\right)}{\partial u(k+r|k)} & =\sum_{i=1}^{n_{x}}\frac{\partial f_{n_{x}}\left({\hat{x}}_{1}\left(k+p-1|k\right),\ldots ,{\hat{x}}_{n_{x}}\left(k+p-1|k\right),u\left(k+p-1|k\right)\right)}{\partial {\hat{x}}_{i}\left(k+p-1|k\right)}\\ \; & \;\;\times \frac{\partial {\hat{x}}_{i}\left(k+p-1|k\right)}{\partial u(k+r|k)}\\ \; & \;+\frac{\partial f_{n_{x}}\left({\hat{x}}_{1}\left(k+p-1|k\right),\ldots ,{\hat{x}}_{n_{x}}\left(k+p-1|k\right),u\left(k+p-1|k\right)\right)}{\partial u(k+p-1|k)}\\ \; & \;\times \frac{\partial u(k+p-1|k)}{\partial u(k+r|k)} \end{array} \end{array}$$

Finally, we can find the predictions of the FP sub-model output using Equation (66)

(86) $$\begin{array}{cc} \frac{\partial {\hat{y}}^{\mathrm{FP}}\left(k+p|k\right)}{\partial u(k+r|k)} & =\sum_{i=1}^{n_{x}}\frac{\partial g({\hat{x}}_{1}\left(k+p|k\right),\ldots ,{\hat{x}}_{n_{x}}\left(k+p|k\right))}{\partial {\hat{x}}_{i}\left(k+p|k\right)}\frac{\partial {\hat{x}}_{i}\left(k+p|k\right)}{\partial u(k+r|k)} \end{array}$$

## 4. Results

### 4.1. Polymerization Process Description

The process under study is a polymerization reactor [43] that is frequently used as a benchmark to assess the usefulness of models and control methods, e.g., [20,27]. This process is characterized by a single input, representing the initiator’s flow rate, denoted as $F_{I}$ (m${}^{3}$ h${}^{-1}$). Likewise, it has a single output, the number average molecular weight (NAMW) (kg kmol${}^{-1}$). Both input and output signals have been appropriately normalized to facilitate the training of neural networks. The scaling is defined as follows: $u=100(F_{I}-{\bar{F}}_{I})$ and $y=0.0001(\mathrm{NAMW}-\bar{\mathrm{NAMW}})$. The values at the nominal operating point are ${\bar{F}}_{I}=0.016783$ and $\bar{\mathrm{NAMW}}=$ 20,000. The polymerization process operates with a sampling time T=1.8 seconds.

Let us note that the predictions determined from the LSTM sub-models are universal, as derived in Section 3.5. Similarly, let us note that the derivatives matrix determined from the LSTM sub-models are universal, as derived in Section 3.8. Hence, it is only necessary to derive specific equations for prediction using the specific first-principle model of the process. Next, we have to derive equations for the derivatives matrix.

### 4.2. First-Principle Model for Polymerization Process and Its Use in MPC

The continuous-time first-principle model of the polymerization process [43] is discreticized using the Euler method. The discrete-time model has the following form:

(87) $$\begin{array}{cc} \;x_{1}\left(k\right) & =T\left(60-10x_{1}\left(k-1\right)\sqrt{x_{2}\left(k-1\right)}\right)+x_{1}\left(k-1\right) \end{array}$$

(88) $$\begin{array}{cc} x_{2}\left(k\right) & =T\left(80u\left(k-1\right)-10.1022x_{2}\left(k-1\right)\right)+x_{2}\left(k-1\right) \end{array}$$

(89) $$\begin{array}{ll} x_{3}\left(k\right) & =T\left(0.0024121x_{1}\left(k-1\right)\sqrt{x_{2}\left(k-1\right)}+0.112191x_{2}\left(k-1\right)-10x_{3}\left(k-1\right)\right)\\ \; & \;+x_{3}\left(k-1\right) \end{array}$$

(90) $$\begin{array}{cc} \;x_{4}\left(k\right) & =T\left(245.978x_{1}\left(k-1\right)\sqrt{x_{2}\left(k-1\right)}-10x_{4}\left(k-1\right)\right)+x_{4}\left(k-1\right) \end{array}$$

(91) $$\begin{array}{cc} \;y^{\mathrm{FP}}\left(k\right) & =\frac{x_{4}\left(k\right)}{x_{3}\left(k\right)} \end{array}$$

where the model parameters are: $p_{1}=60T$, $p_{2}=-10T$, $p_{3}=80T$, $p_{4}=-10.1022T+1$, $p_{5}=0.0024121T$, $p_{6}=0.112191T$, $p_{7}=-10T+1$, $p_{8}=245.978T$, $p_{9}=-10T+1$.

It is important to note that to emulate the imperfections and inaccuracies of the FP model, we introduced a 20 percent increase to the gain of the model during the simulation experiments, i.e.,

(92) $$y_{\mathrm{disturbed}}^{\mathrm{FP}}\left(k\right)=1.2y^{\mathrm{FP}}\left(k\right)=1.2\frac{x_{4}\left(k\right)}{x_{3}\left(k\right)}$$

For the PIHNN model used in our MPC algorithm, we have to derive equations for the prediction using the specific FP model of the considered benchmark system and the general rules formulated in Section 3.6. They will allow to calculate the predicted trajectory ${\hat{y}}^{\mathrm{traj}}\left(k\right)$, as defined by Equation (34). We start with determining prediction equations when p=1. From Equations (87)–(91), we obtain

(93) $$\begin{array}{cc} \;{\hat{x}}_{1}\left(k+1|k\right) & =p_{1}+p_{2}x_{1}\left(k\right)\sqrt{x_{2}\left(k\right)}+x_{1}\left(k\right)+\nu_{1}\left(k\right) \end{array}$$

(94) $$\begin{array}{cc} \;{\hat{x}}_{2}\left(k+1|k\right) & =p_{3}u\left(k|k\right)+p_{4}x_{2}\left(k\right)+\nu_{2}\left(k\right) \end{array}$$

(95) $$\begin{array}{cc} {\hat{x}}_{3}\left(k+1|k\right) & =p_{5}x_{1}\left(k\right)\sqrt{x_{2}\left(k\right)}+p_{6}x_{2}\left(k\right)+p_{7}x_{3}\left(k\right)+\nu_{3}\left(k\right) \end{array}$$

(96) $$\begin{array}{cc} \;{\hat{x}}_{4}\left(k+1|k\right) & =p_{8}x_{1}\left(k\right)\sqrt{x_{2}\left(k\right)}+p_{9}x_{4}\left(k\right)+\nu_{4}\left(k\right) \end{array}$$

(97) $$\begin{array}{cc} \;{\hat{y}}^{\mathrm{FP}}\left(k+1|k\right) & =\frac{{\hat{x}}_{4}\left(k+1|k\right)}{{\hat{x}}_{3}\left(k+1|k\right)}+d\left(k\right) \end{array}$$

The state and output disturbances are derived from the general Equations (67)–(69), respectively, which gives

(98) $$\begin{array}{cc} \;\nu_{1}\left(k\right) & =x_{1}\left(k\right)-p_{1}+p_{2}x_{1}\left(k-1\right)\sqrt{x_{2}\left(k-1\right)}+x_{1}\left(k-1\right) \end{array}$$

(99) $$\begin{array}{cc} \;\nu_{2}\left(k\right) & =x_{2}\left(k\right)-p_{3}u\left(k-1\right)+p_{4}x_{2}\left(k-1\right) \end{array}$$

(100) $$\begin{array}{cc} \nu_{3}\left(k\right) & =x_{3}\left(k\right)-p_{5}x_{1}\left(k-1\right)\sqrt{x_{2}\left(k-1\right)}+p_{6}x_{2}\left(k-1\right)+p_{7}x_{3}\left(k-1\right) \end{array}$$

(101) $$\begin{array}{cc} \;\nu_{4}\left(k\right) & =x_{4}\left(k\right)-p_{8}x_{1}\left(k-1\right)\sqrt{x_{2}\left(k-1\right)}+p_{9}x_{4}\left(k-1\right) \end{array}$$

(102) $$\begin{array}{cc} \;d(k) & =y\left(k\right)-\frac{x_{4}\left(k\right)}{x_{3}\left(k\right)} \end{array}$$

Next, we find the equations for state and output predictions for p=2,⋯,N

(103) $$\begin{array}{cc} \;{\hat{x}}_{1}\left(k+p|k\right) & =p_{1}+p_{2}{\hat{x}}_{1}\left(k+p-1|k\right)\sqrt{{\hat{x}}_{2}\left(k+p-1|k\right)}+{\hat{x}}_{1}\left(k+p-1|k\right)+\nu_{1}\left(k\right) \end{array}$$

(104) $$\begin{array}{cc} {\hat{x}}_{2}\left(k+p|k\right) & =p_{3}u\left(k+p-1|k\right)+p_{4}{\hat{x}}_{2}\left(k+p-1|k\right)+\nu_{2}\left(k\right) \end{array}$$

(105) $$\begin{array}{cc} {\hat{x}}_{3}\left(k+p|k\right) & =p_{5}{\hat{x}}_{1}\left(k+p-1|k\right)\sqrt{x_{2}\left(k+p-1|k\right)}+p_{6}x_{2}\left(k+p-1|k\right)\\ \; & \;+p_{7}{\hat{x}}_{3}\left(k+p-1|k\right)+\nu_{3}\left(k\right) \end{array}$$

(106) $$\begin{array}{cc} \;{\hat{x}}_{4}\left(k+p|k\right) & =p_{8}{\hat{x}}_{1}\left(k+p-1|k\right)\sqrt{{\hat{x}}_{2}\left(k+p-1|k\right)}+p_{9}{\hat{x}}_{4}\left(k+p-1|k\right)+\nu_{4}\left(k\right) \end{array}$$

(107) $$\begin{array}{cc} \;{\hat{y}}^{\mathrm{FP}}\left(k+p|k\right) & =\frac{{\hat{x}}_{4}\left(k+p|k\right)}{{\hat{x}}_{3}\left(k+p|k\right)}+d\left(k\right) \end{array}$$

Using the above predictions generated by the FP model, we have to determine derivatives of the predicted trajectory of the controlled variable with respect to the trajectory of the manipulated variable, i.e., the derivative matrix H(k), as defined by Equation (38). For this purpose, we use the general rules formulated in Section 3.9. We consider Equations (81) and (82) and we obtain

(108) $$\begin{array}{cc} \frac{\partial {\hat{x}}_{1}\left(k+1|k\right)}{\partial u(k+r|k)} & =0 \end{array}$$

(109) $$\begin{array}{cc} \;\frac{\partial {\hat{x}}_{2}\left(k+1|k\right)}{\partial u(k+r|k)} & =80T\frac{\partial u(k|k)}{\partial u(k+r|k)} \end{array}$$

(110) $$\begin{array}{cc} \frac{\partial {\hat{x}}_{3}\left(k+1|k\right)}{\partial u(k+r|k)} & =0 \end{array}$$

(111) $$\begin{array}{cc} \frac{\partial {\hat{x}}_{4}\left(k+1|k\right)}{\partial u(k+r|k)} & =0 \end{array}$$

Equation (83) allows us to express the output derivatives as

(112) $$\begin{array}{cc} \frac{\partial {\hat{y}}^{\mathrm{FP}}\left(k+p|k\right)}{\partial u(k+r|k)} & =0 \end{array}$$

Finally, we use Equations (84)–(86) to determine the state variable and output derivatives, respectively

(113) $$\begin{array}{c} \begin{array}{cc} \frac{\partial {\hat{x}}_{1}\left(k+p|k\right)}{\partial u(k+r|k)} & =p_{2}\frac{\partial {\hat{x}}_{1}\left(k+p-1|k\right)}{\partial u(k+r|k)}\sqrt{{\hat{x}}_{2}\left(k+p-1|k\right)}-0.5p_{2}{\hat{x}}_{1}\left(k+p-1|k\right)\\ \; & \;\times {\left({\hat{x}}_{2}\left(k+p-1|k\right)\right)}^{-2}\frac{\partial {\hat{x}}_{2}\left(k+p-1|k\right)}{\partial u(k+r|k)}+\frac{{\hat{x}}_{1}\left(k+p-1|k\right)}{\partial u(k+r|k)} \end{array} \end{array}$$

(114) $$\begin{array}{cc} \;\frac{\partial {\hat{x}}_{2}\left(k+p|k\right)}{\partial u(k+r|k)} & =p_{3}\frac{\partial u(k+p-1|k)}{\partial u(k+r|k)}+p_{4}\frac{{\hat{x}}_{2}\left(k+p-1|k\right)}{\partial u(k+r|k)} \end{array}$$

(115) $$\begin{array}{c} \begin{array}{cc} \frac{\partial {\hat{x}}_{3}\left(k+p|k\right)}{\partial u(k+r|k)} & =p_{5}\frac{\partial {\hat{x}}_{1}\left(k+p-1|k\right)}{\partial u(k+r|k)}\sqrt{{\hat{x}}_{2}\left(k+p-1|k\right)}-0.5p_{5}{\hat{x}}_{1}\left(k+p-1|k\right)\\ \; & \;\times {\left({\hat{x}}_{2}\left(k+p-1|k\right)\right)}^{-2}\frac{\partial {\hat{x}}_{2}\left(k+p-1|k\right)}{\partial u(k+r|k)}+p_{6}\frac{{\hat{x}}_{2}\left(k+p-1|k\right)}{\partial u(k+r|k)}\\ \; & \;+p_{7}\frac{{\hat{x}}_{3}\left(k+p-1|k\right)}{\partial u(k+r|k)} \end{array} \end{array}$$

(116) $$\begin{array}{c} \begin{array}{cc} \frac{\partial {\hat{x}}_{4}\left(k+p|k\right)}{\partial u(k+r|k)} & =p_{8}\frac{\partial {\hat{x}}_{1}\left(k+p-1|k\right)}{\partial u(k+r|k)}\sqrt{{\hat{x}}_{2}\left(k+p-1|k\right)}-0.5p_{8}{\hat{x}}_{1}\left(k+p-1|k\right)\\ \; & \;\times {\left({\hat{x}}_{2}\left(k+p-1|k\right)\right)}^{-2}\frac{\partial {\hat{x}}_{2}\left(k+p-1|k\right)}{\partial u(k+r|k)}+p_{9}\frac{{\hat{x}}_{4}\left(k+p-1|k\right)}{\partial u(k+r|k)} \end{array} \end{array}$$

(117) $$\begin{array}{cc} \frac{\partial {\hat{y}}^{\mathrm{FP}}\left(k+p|k\right)}{\partial u(k+r|k)} & =\frac{1}{{\hat{x}}_{3}{\left(k+p|k\right)}^{2}}\left(\frac{\partial {\hat{x}}_{4}\left(k+p|k\right)}{\partial u(k+r|k)}{\hat{x}}_{3}\left(k+p|k\right)-{\hat{x}}_{4}\left(k+p|k\right)\frac{\partial {\hat{x}}_{3}\left(k+p|k\right)}{\partial u(k+r|k)}\right) \end{array}$$

for all p=2,…,N and $r=0,\ldots ,N_{u}-1$.

### 4.3. LSTM Model for Polymerization Process

Two separate training datasets have been collected from the simulated process (i.e., from simulation of the continuous-time first-principle models), for different operating conditions, as follows:

1. dataset 1 has been collected for the range of the manipulated variable $0.003<F_{I}<0.0129$, which results in the controlled variable $2.78\times 10^{4}<\mathrm{NAMW}<4.55\times 10^{4}$.
2. dataset 2 has been collected for $0.05<F_{I}<0.06$ which results in $1.41\times 10^{4}<\mathrm{NAMW}<1.54\times 10^{4}$.

The datasets were then used to train two LSTM models, denoted thereafter as LSTM1 and LSTM2. Both models have been trained with the same parameters:

- the number of neurons $n_{N}=7$;
- the order of dynamics $n_{A}=0,n_{B}=1$.

LSTM models have been trained in MATLAB on a PC equipped with an Nvidia GeForce 970 GTX GPU, an Intel i5-3450 CPU and 16 GB of RAM. We have employed the Adam optimization algorithm with a learning rate of 0.001 and a maximum of 1000 training epochs.

### 4.4. Modeling Quality of LSTM and FP Models

**Figure 5:**
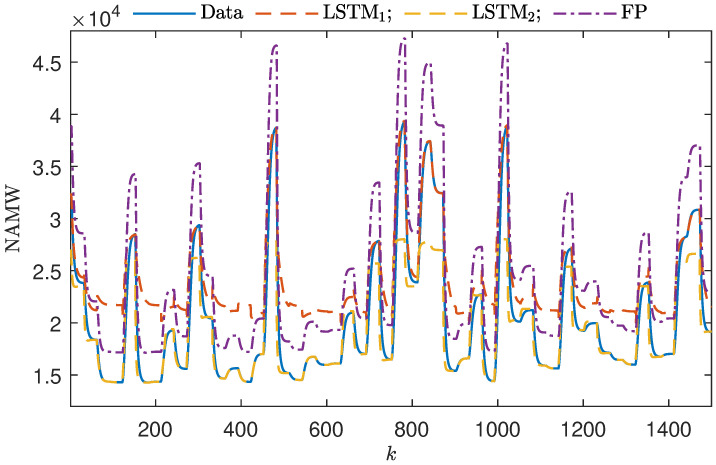
A total of 1000 samples of the validation dataset vs. outputs of two local LSTM sub-models and the FP model with an incorrect gain.

The modeling quality of all sub-models developed for the polymerization process can be compared in Figure 5. In this comparison, we can see the individual outputs of all sub-models when operating independently with the test dataset. LSTM1, trained predominantly with data featuring large NAMW values, unsurprisingly demonstrates exceptional performance when dealing with such high NAMW values. However, the model’s capability to provide correct outputs diminishes when it encounters data not present in the training dataset. Conversely, LSTM2, trained with low NAMW values, excels when the NAMW values are indeed low. However, it exhibits subpar performance when attempting to model high NAMW values. Notably, in the FP model with the increased gain performs poorly across the entire range of NAMW values.

### 4.5. Development of PIHNN Models

Once all the sub-models have been prepared, the next step to design the PIHNN model is to develop the DF block. Various membership function shapes have been tested, i.e.:

- PIHNN model ver. 1—initial trapezoidal functions;
- PIHNN model ver. 2—optimized trapezoidal functions;
- PIHNN model ver. 3—initial trapezoidal functions;
- PIHNN model ver. 4—optimized trapezoidal functions;
- PIHNN model ver. 5—initial trapezoidal functions;
- PIHNN model ver. 6—optimized trapezoidal functions.

**Figure 6:**
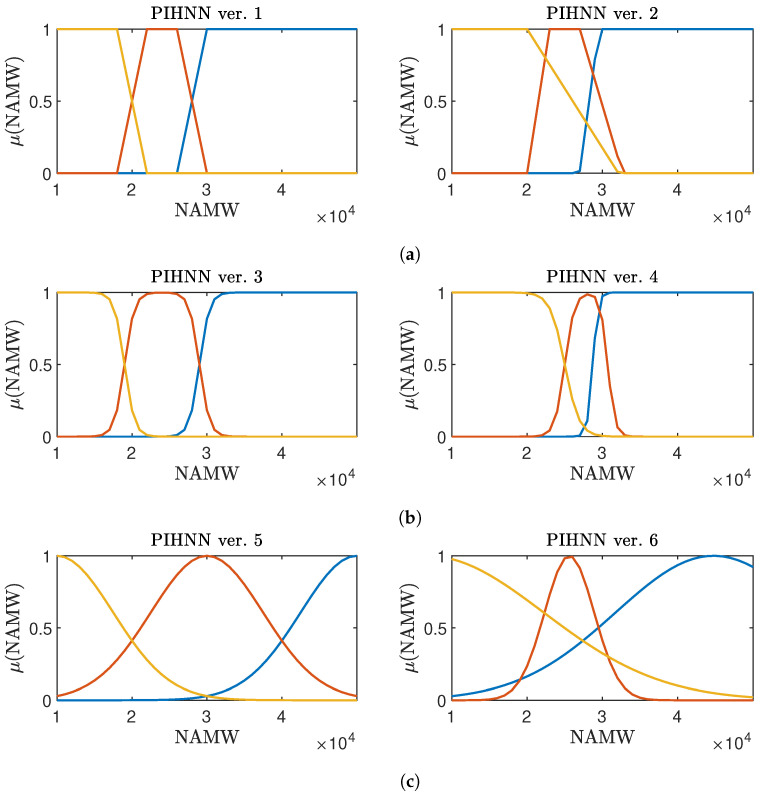
Membership functions for considered fuzzy PIHNN models: fuzzy set 1 (blue), fuzzy set 2 (orange), fuzzy set 3 (yellow). (**a**) Initial (**left**) and optimized (**right**) trapezoidal membership functions; (**b**) initial (**left**) and optimized (**right**) sigmoidal membership functions; (**c**) initial (**left**) and optimized (**right**) Gauss membership functions.

The membership functions are depicted in Figure 6. Our understanding of the sub-models has guided the initial choices of these shapes. The plots display fuzzified variable values along the horizontal axis, specifically representing the NAMW output of the polymerization reactor. Along the vertical axis, one can find the membership function values. Each membership function corresponds to a particular model. LSTM1, which was trained on data with large NAMW values, is most effective when dealing with large NAMW values. The blue membership functions on the plot indicate the range of NAMW values for which prioritizing the use of the LSTM1 model is recommended. LSTM2, characterized by yellow membership functions, is best suited for NAMW values close to the data in its training set, which primarily includes small values of NAMW. In scenarios where NAMW values fall outside the data ranges of both training sets, the most reliable choice is to utilize the FP model, represented by orange membership functions. Once the initial shapes have been determined, the subsequent step involves utilizing an optimization procedure to fine-tune these shapes. The procedure starts with initial membership function shapes, using Levenberg–Marquardt to minimize the overall error of the PIHNN model.

### 4.6. PIHNN Modeling Quality

**Figure 7:**
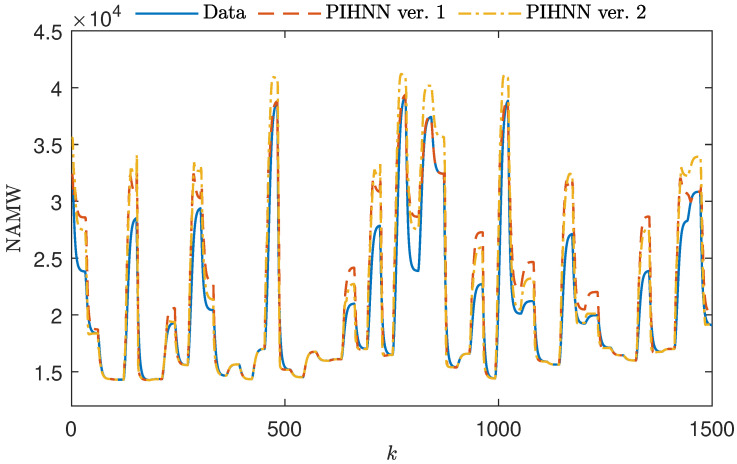
A total of 1000 samples of the validation dataset vs. the output of initial and optimized fuzzy PIHNN structures with trapezoidal MFs (PIHNN models ver. 1 and ver. 2).

**Figure 8:**
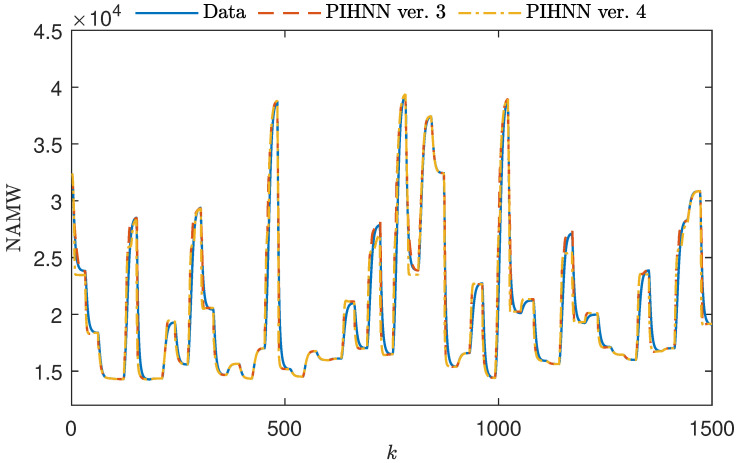
A total of 1000 samples of the validation dataset vs. the output of initial and optimized fuzzy PIHNN structures with sigmoid MFs (PIHNN models ver. 3 and ver. 4).

**Figure 9:**
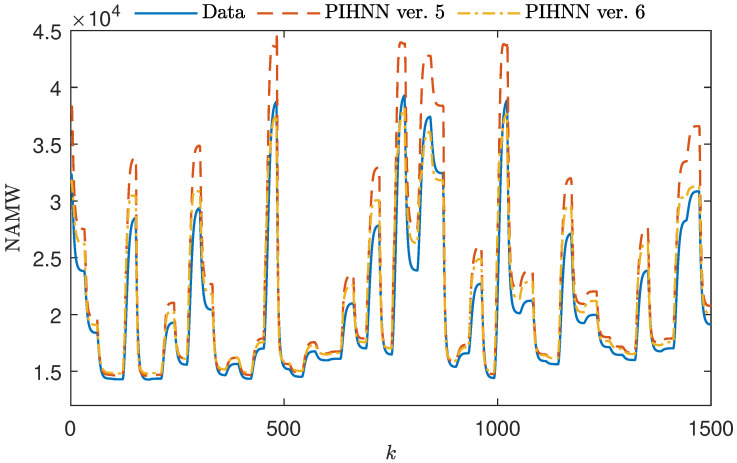
A total of 1000 samples of the validation dataset vs. the output of initial and optimized fuzzy PIHNN structures with Gauss MFs (PIHNN models ver. 5 and ver. 6).

The results of the polymerization reactor modeling experiments are presented in Figure 7, Figure 8 and Figure 9. These figures illustrate the initial 1500 steps of the simulation. Each figure showcases the outputs of two PIHNN models: one with the initial membership function shapes (orange) and the other with optimized (yellow) membership function shapes. These results are compared to the data from the test set. Figure 7 presents the use of the most straightforward decision blocks with trapezoidal membership functions. Even this simplest approach enables the PIHNN model to outperform individual sub-models. The initial shape of the membership function allows the PIHNN structure to represent the data effectively for both small and large values of NAMW. In cases with intermediate values of NAMW, the PIHNN model averages the outputs of the sub-models, while model output still exhibits some deviation from the test data, there is a clear improvement over the FP model. The model with a tuned shape has lower error overall; however, it tends to have poorer modeling quality for both large and small values of NAMW in comparison to the LSTM sub-models.

Figure 8 illustrates the utilization of sigmoidal membership functions in the DF block of the PIHNN model. Here, the sigmoidal shape allows the PIHNN to excel in modeling small, large, and intermediate NAMW values. Importantly, the tendency to average out intermediate NAMW values, as previously observed with the trapezoidal DF model, has been eliminated with the sigmoidal DF PIHNN model. Adopting sigmoidal functions has resulted in highly accurate modeling of medium NAMW values. When comparing the output signals of the models with the initial and tuned shapes of the membership functions, they exhibit minimal differences, with only slight variations noticeable for intermediate values of NAMW.

Finally, Figure 9 presents the utilization of Gaussian membership functions in a DF block of PIHNN model. Here, one can observe that the Gaussian decision model tends to average the values of the three sub-models across the entire spectrum of NAMW variability. This effect is particularly evident in the model with the initial shape of the membership function, where, for large values of NAMW, the model noticeably diverges from the data. As a result, for large NAMW values, PIHNN gives worse results than the independent LSTM1 submodel. Low and intermediate NAMW values are subject to much lower modeling errors. Although optimizing the shape mitigated this averaging effect somewhat, the model’s output still exhibits relatively large errors.

### 4.7. Validation of MPC Algorithms Using PIHNN Models

The PIHNN model, in six different versions, has been implemented in MPC algorithms. We compare the results obtained from two types of controllers: one with nonlinear optimization (MPC-NO) and the second one recommended in this work, involving linearization around the prediction trajectory (MPC-NPLPT). Table 1 compares the control errors determined for these controllers. First, it is worth noting that the best control quality is achieved for models utilizing DF with Gaussian function shapes. Models employing trapezoidal functions exhibited slightly higher errors, while the poorest performance was observed in models with sigmoidal-shaped functions. This observation may seem counter-intuitive, considering that models with sigmoidal membership functions have smaller modeling errors compared to models with Gaussian ones. It is important to stress that the shape of the closed-loop output trajectory with the MPC controller is affected not only by the quality of the model used but also by the feedback mechanism. Even though Gaussian models exhibit a higher error rate, their inherent averaging characteristic enhances the performance of the MPC controller when coupled with feedback.

Secondly, Table 1 demonstrates that the MPC-NPLPT controller generally yields slightly higher error values than the MPC-NO one when utilizing the same PIHNN model for prediction. This result is not surprising, as MPC-NPLPT employs a linearized model. During linearization, some of the information present in the nonlinear model is simplified or lost. The exception here is PIHNN model ver. 5, where MPC-NPLPT algorithm provides better controller performance. This may be attributed to chance where the simplifications happened to benefit the controller’s performance in this specific case. However, it is worth noting that the error differences between MPC-NO and MPC-NPLT controllers are minimal for each type of PIHNN model, and both types of controllers work very well.

Table 2 compares the average time required by each MPC controller for control calculations. The computations have been conducted on a PC, and since it is not a real-time system, results may vary on different PCs. Therefore, the results are presented as percentages. The longest time recorded for MPC-NO with PIHNN model ver. 3, which amounted to 140 ms, is considered as 100%. The table reveals that the implementation of the online linearization-based MPC controller significantly reduced the calculation time required, resulting in a 4–5 times decrease compared to nonlinear controllers.

**Figure 10:**
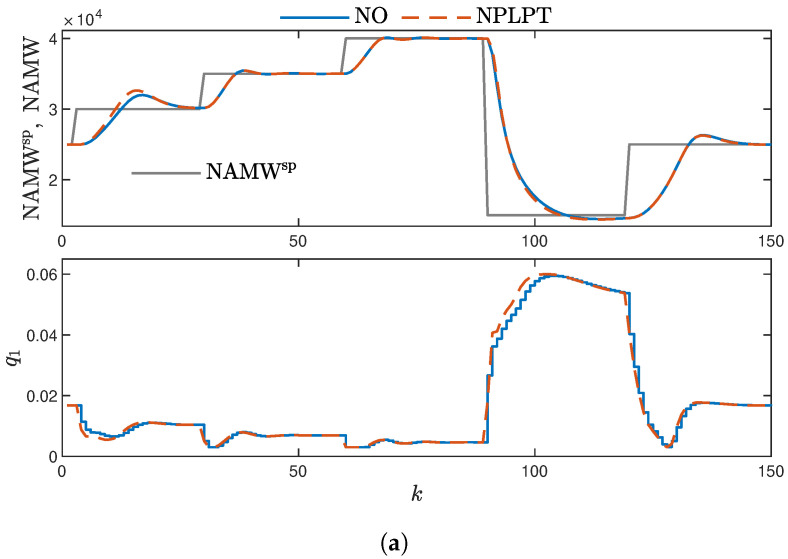

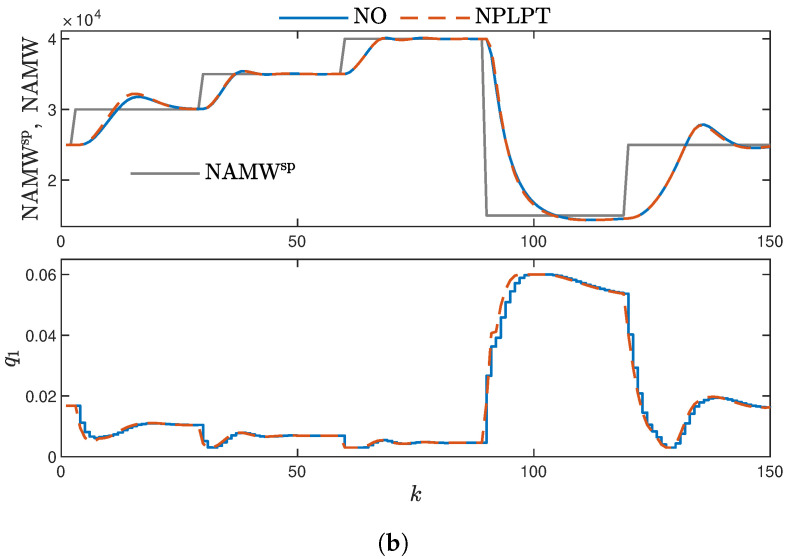
MPC with trapezoidal membership function shapes. (**a**) MPC-NO and MPC-NPLPT controllers using PIHNN model ver. 1; (**b**) MPC-NO and MPC-NPLPT controllers using PIHNN model ver. 2.

The results are also visually presented. In Figure 10, one can observe the performance of the MPC algorithm with a DF employing trapezoidal functions. The output signals for the PIHNN model with the initial function shape are swift without overshoot for both low and high values of NAMW. However, for intermediate NAMW values, there is a slightly larger overshoot, and the settling time is extended. The signals are quite similar in the case of DF with a tuned function shape, but there is a greater overshoot for intermediate NAMW values. Additionally, it is worth noting that the results obtained for the MPC-NO controller are practically indistinguishable from those for the MPC-NPLT one.

**Figure 11:**
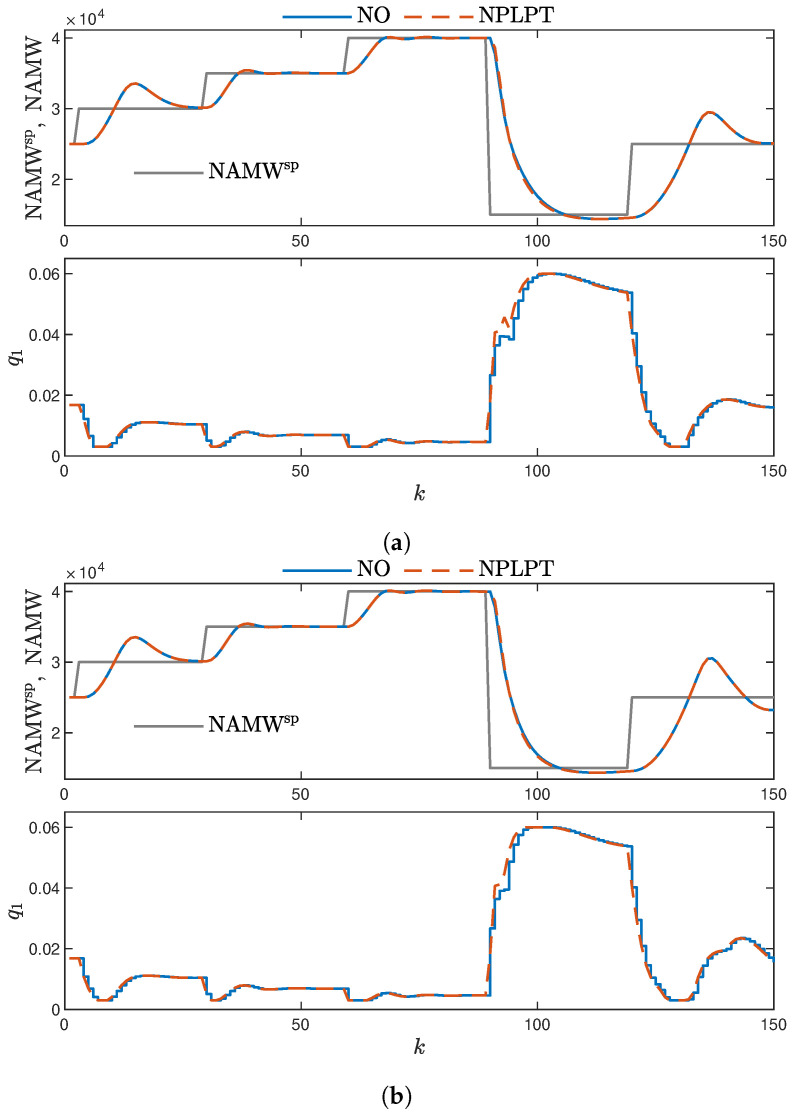
MPC controllers with sigmoidal membership function shapes. (**a**) MPC-NO and MPC-NPLPT controllers using PIHNN model ver. 3; (**b**) MPC-NO and MPC-NPLPT controllers using PIHNN model ver. 4.

Figure 11 illustrates the results for sigmoidal membership functions. Here, we can observe that the overshoot becomes more pronounced, particularly for intermediate NAMW values, especially when considering the set-point ${\mathrm{NAMW}}^{\mathrm{sp}}=2.5\times 10^{4}$.

**Figure 12:**
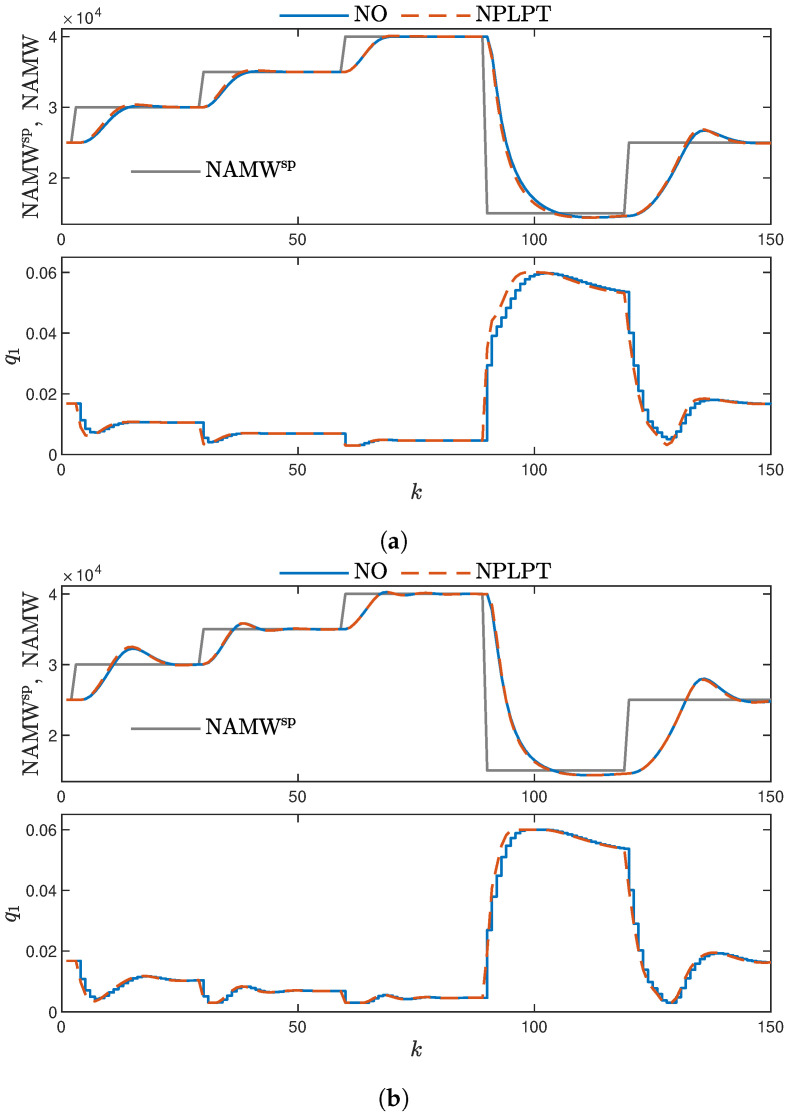
MPC controllers with Gaussian membership function shapes. (**a**) MPC-NO and MPC-NPLPT controllers using PIHNN model ver. 5; (**b**) MPC-NO and MPC-NPLPT controllers using PIHNN model ver. 6.

The final Figure 12 displays the results of applying Gaussian membership functions. These results are characterized by the shortest settling time and the smallest overshoot. Notably, the controller exhibits excellent performance for average values of NAMW. This observation leads to the conclusion that the averaging nature of Gaussian functions, as seen earlier in the modeling phase (Figure 9, positively impacts the controller’s performance when using the model in the MPC scheme. For NAMW values within the range of $2\times 10^{4}$ to $3\times 10^{4}$, the FP model significantly impacts PIHNN performance. As mentioned, the FP model is imperfect, featuring an increased gain of 20%.

## 5. Conclusions

This work defines a new PIHNN model structure that combines the first-principle process description and data-driven neural sub-models using a specialized data fusion block that relies on fuzzy logic. We consider a very practical case when the available first-principle model is imperfect and the data cannot be measured in the complete range of process operation. By combining an imperfect physical model with data obtained from an incomplete range of operations, we have developed a hybrid model that significantly improves performance across the entire range of signal variability. Secondly, this work develops a computationally efficient MPC controller for the PIHNN model. We show the efficacy of the PIHNN model and the resulting MPC controller for a simulated polymerization benchmark. We study the efficiency of different data fusion fuzzy blocks and their impact on model accuracy. We recommend tuning, i.e., optimizing the fuzzy membership functions, greatly improving model accuracy. Finally, we show that the described MPC controller based on the PIHNN model gives excellent results. Namely, the obtained control quality is very similar to that possible in MPC relying on nonlinear optimization while its calculation time is a few times shorter. In our future work, we plan to develop a methodology for designing PIHNN structures tailored to processes with multiple inputs and outputs. Additionally, it is interesting to check the impact of employing various decision model types within the data fusion block on PIHNN modeling quality.

## Figures and Tables

**Table 1:** Control errors of MPC algorithms with different PIHNN models.

Model Type	MPC-NO	MPC-NPLPT
PIHNN ver. 1	3.051	3.116
PIHNN ver. 2	3.031	3.095
PIHNN ver. 3	3.205	3.263
PIHNN ver. 4	3.220	3.290
PIHNN ver. 5	2.935	2.741
PIHNN ver. 6	2.965	3.020

**Table 2:** Average execution time of MPC algorithms with different PIHNN models.

Model Type	MPC-NO	MPC-NPLPT
PIHNN ver. 1	94.4%	22.4%
PIHNN ver. 2	97.2%	23.1%
PIHNN ver. 3	100.0%	21.0%
PIHNN ver. 4	97.2%	23.1%
PIHNN ver. 5	89.3%	24.5%
PIHNN ver. 6	93.7%	23.1%

## Data Availability

On request from the authors.
